# Stiffness reconstruction methods for MR elastography

**DOI:** 10.1002/nbm.3935

**Published:** 2018-05-18

**Authors:** Daniel Fovargue, David Nordsletten, Ralph Sinkus

**Affiliations:** ^1^ Imaging Sciences & Biomedical Engineering King's College London London UK; ^2^ Inserm U1148, LVTS University Paris Diderot, University Paris 13 Paris 75018 France

**Keywords:** inverse problem, MR elastography, reconstruction, review, shear‐modulus tissue stiffness

## Abstract

Assessment of tissue stiffness is desirable for clinicians and researchers, as it is well established that pathophysiological mechanisms often alter the structural properties of tissue. Magnetic resonance elastography (MRE) provides an avenue for measuring tissue stiffness and has a long history of clinical application, including staging liver fibrosis and stratifying breast cancer malignancy. A vital component of MRE consists of the reconstruction algorithms used to derive stiffness from wave‐motion images by solving inverse problems. A large range of reconstruction methods have been presented in the literature, with differing computational expense, required user input, underlying physical assumptions, and techniques for numerical evaluation. These differences, in turn, have led to varying accuracy, robustness, and ease of use. While most reconstruction techniques have been validated against in silico or in vitro phantoms, performance with real data is often more challenging, stressing the robustness and assumptions of these algorithms. This article reviews many current MRE reconstruction methods and discusses the aforementioned differences. The material assumptions underlying the methods are developed and various approaches for noise reduction, regularization, and numerical discretization are discussed. Reconstruction methods are categorized by inversion type, underlying assumptions, and their use in human and animal studies. Future directions, such as alternative material assumptions, are also discussed.

**Abbreviations used**
CDI: Curl‐based direct inversion
DI: Direct inversion
FEM: Finite element method
LFE: Local frequency estimation
MDEV: Multifrequency dual elastovisco
MRE: Magnetic resonance elastography
TWE: Traveling wave expansion.

## 1 INTRODUCTION

Tissue stiffness is often affected both directly and indirectly by disease, with common examples being fibrosis, tumor growth, and inflammation. As tissue becomes fibrotic, collagen density increases in the extracellular matrix, leading to a macroscale effect on stiffness.^1^, ^2^ During tumor growth, angiogenesis, increased cellular stiffness, and compaction of surrounding tissue can combine to alter material properties.^3^, ^4^ The significant changes in hardness often accompanying disease have historically led to palpation being used as an early tool for the detection of certain diseases. While still invaluable for initial screening, palpation is qualitative, limited to superficial regions of the body, and often most applicable in advanced stages of disease.

Elastography is an imaging technique that infers stiffness by examining a tissue's response to some excitation. This excitation, often externally applied, leads to deformation in the tissue, which can be measured by medical imaging methods such as magnetic resonance (MR) or ultrasound (US). These measured deformations can be used to reconstruct stiffness values by solving equations based on a chosen tissue model that relates these quantities. The calculated values of stiffness (typically the shear modulus) are similar to what is felt during palpation.^5^ However, elastography, besides being quantitative, has the added benefits of being able to examine deeper in the body, potentially detecting small changes in stiffness present in earlier stages of disease, and measuring additional viscous properties of the tissue.^6^ Furthermore, the shear modulus has been seen to vary more over various healthy and diseased tissue types than the bulk modulus or other imaging modalities such as *T*_1_ relaxation.^7^

The first experiments in elastography began with US imaging.^8^, ^9^, ^10^, ^11^ The low cost and wide availability of US continues to make this a popular elastography imaging method.^12^ Later, MR methods for elasticity imaging were developed,^13^, ^14^, ^15^ with advantages including a full 3D image stack of the tissue displacements and large, movable, and deeper fields of view.^7^, ^12^ For a history of US and MR elastography (MRE), see Sarvazyan et al.^16^ Elastography has also been performed with optical coherence tomography (OCT), where advantages include high resolution and smaller scale imaging,^17^, ^18^, ^19^, ^20^, ^21^ X‐ray,^22^ and mechanical imaging,^23^ which measures surface stress patterns to infer internal structure.

**Figure 1:**
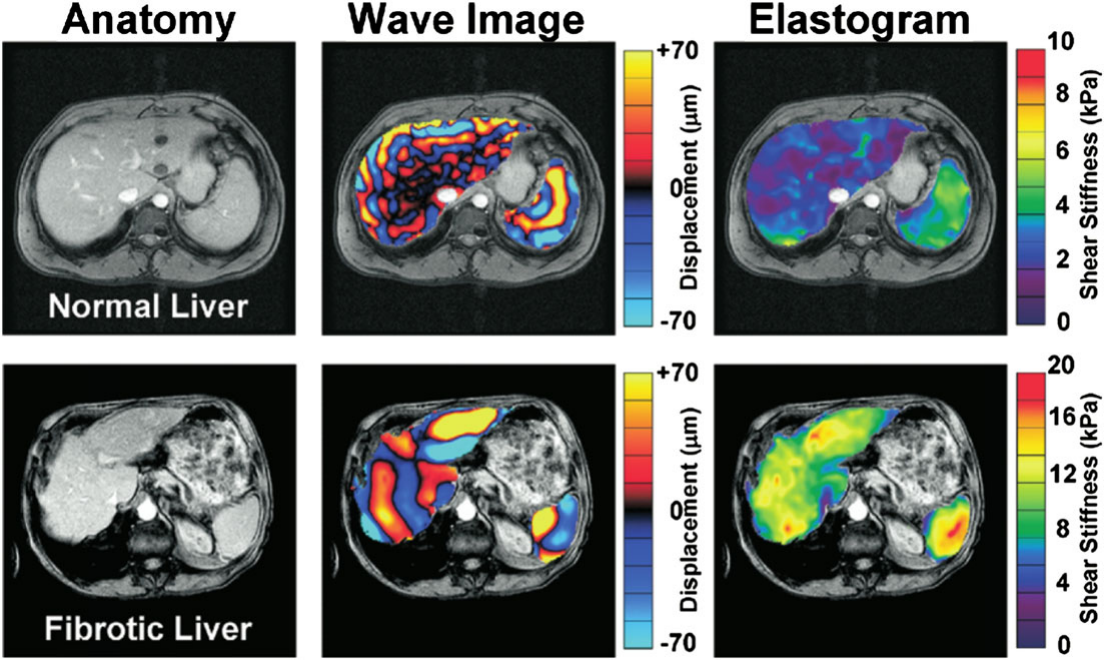
Anatomical, wave, and stiffness images of normal and fibrotic liver. While little difference is apparent in the anatomical images, large differences are seen in the wave data and reconstructed stiffness. Also clearly demonstrated is the relationship between increased wavelength and increased stiffness (Reprinted from Yin et al^24^ with permission from Elsevier)

MRE has demonstrated success in many areas. Investigations into liver have revealed that MRE is capable of staging liver fibrosis^24^, ^25^ (Figure 1) with greater separation than other modalities, including US elastography and MR diffusion‐weighted imaging.^26^, ^27^ MRE has also seen success in differentiating benign and malignant tumors in breast^28^ and liver tumors from surrounding healthy and fibrotic tissue^29^ by their viscoelastic characteristics. MRE is able to image waves in the brain, leading to research on stiffness changes due to Alzheimer's,^30^, ^31^ Parkinson's,^32^ and multiple sclerosis^33^, ^34^ (as opposed to US elastography, which has only been used to measure brain stiffness in surgical contexts^35^, ^36^.

**Figure 2:**
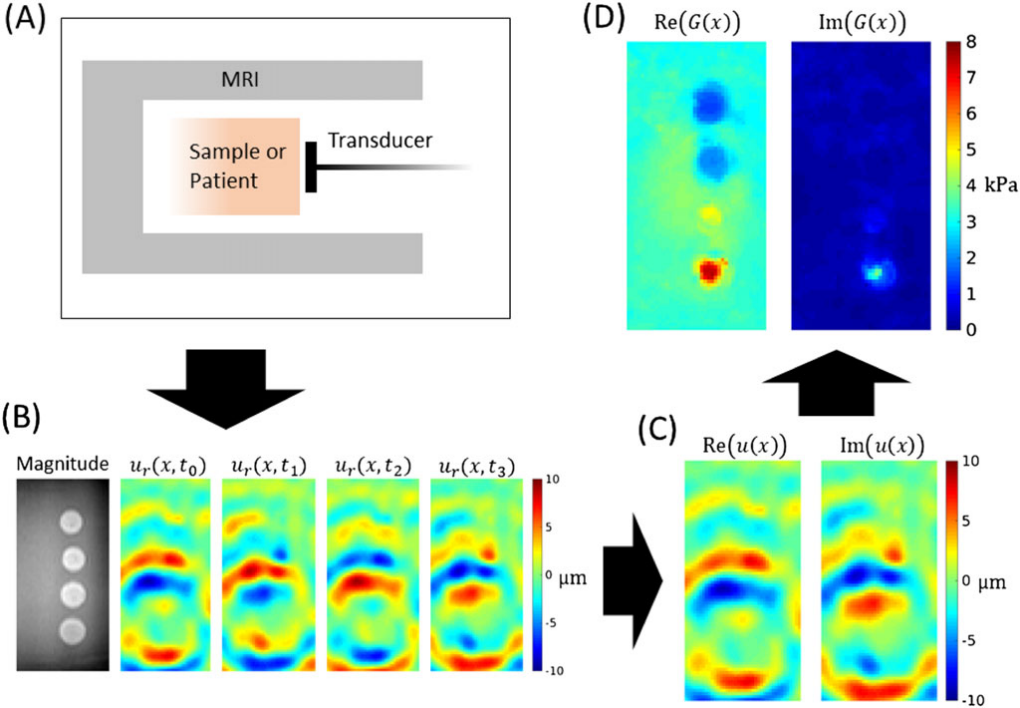
An illustration of a typical process of harmonic MRE. A, The patient or object is subjected to a single frequency vibration while in the MR scanner. Here, data collected from MRE performed on a CIRS 049 elastography phantom (CIRS Inc., Norfolk, VA, USA) are shown. B, During phase contrast imaging, the complex‐valued k‐space data are collected and constructed into a magnitude image and 3D displacement field images at several time points along the cycle of the vibration. C, The real‐valued time‐dependent displacement data are converted by Fourier transform to complex‐valued steady‐state wave data. D, A reconstruction algorithm solves an inverse problem to compute the complex shear modulus.^38^ The shape and differing stiffness values of the inclusions in the phantom are readily apparent in the reconstruction, whereas in the MR magnitude image in B the inclusions appear very similar. The imaginary part of the shear modulus in this phantom should be zero throughout. This appears to be the case with this reconstructed image, besides the small spike in the stiffest inclusion. This stiff region corresponds to a long wavelength and this highlights an important balance in MRE. For longer wavelengths, noise becomes a more dominant factor during calculations, for some reconstruction methods, potentially leading to more error in the resulting stiffness

Within MRE there are many differing implementations, where considerations include the type of tissue excitation, the hardware required to produce the excitation, acquisition methods, and reconstruction methods. Three common tissue excitation techniques across elastography are quasi‐static deformations, transient waves due to singular pulses, and harmonic wave motion due to a single frequency vibration; however, the most common technique in MRE is harmonic.^37^ The process of harmonic MRE (Figure 2) works by inducing periodically steady waves in a region of interest (ROI) in a patient or object, performing phase‐contrast MR imaging, and, from the complex‐valued k‐space data, constructing displacement data representing wave motion within the ROI. Finally, a reconstruction method is applied to calculate the underlying stiffness that modulated the measured wave behavior. Therefore, elastography reconstruction is considered an inverse problem. Typically, due to the small displacements involved, the linear elasticity or linear viscoelasticity equations are used to relate the displacements and stiffness, and hence it is these equations that are inverted. The resulting image of elasticity is usually of the same resolution as the displacement images and is sometimes called an *elastogram*.

MRE reconstruction algorithms have been implemented in many ways, with different underlying assumptions and employing varying processes for the inverse solution. Understanding these differences is important, as they effect uniqueness, accuracy, robustness, ease of use, and required computation time. This article discusses these effects while comparing and summarizing MRE reconstruction methods and associated studies currently present in the literature. The theory underlying MRE reconstruction is presented and the applicability of MRE in various human and animal studies is described.

In Section 2, theory and background that is common across many MRE reconstruction methods is presented. Section 3 contains descriptions and categorizations of various methods and includes some mathematical underpinnings and references to reconstruction usage in other studies. A discussion and conclusion are provided in Section 4, highlighting the scientific and clinical value of MRE as well as some of the key challenges in reconstruction.

## 2 THEORY

In harmonic MRE, a transducer is placed against a patient and produces single‐frequency waves (typically 30–90 Hz) that move through an ROI. Due to the small displacements associated with these waves, the linear elasticity or viscoelasticity equations are typically assumed to govern the tissue deformation. An isotropic assumption is also typically made of tissue during MRE, which is usually considered reasonable depending on the tissue type. The isotropic linear elastic equations contain three material parameters. The first is the density, *ρ*, and common pairs for the remaining two include the following: (i) the two Lamé parameters, λ and *G*(the latter is also called the shear modulus); and (ii) the Young's modulus, *E*, and Poisson's ratio, *ν*. As materials approach incompressibility, the following occur: *ν*→0.5, λ→*∞*, and *E*→3*G*. For tissue that is nearly incompressible, knowing the shear modulus is therefore essentially equivalent to knowing the Young's modulus.

The wave displacements are constructed from complex‐valued k‐space MRI data. These data are captured at several time points over the period of the wave (four or eight time points is common). Occasionally, even at this stage, qualitative conclusions can be made regarding the underlying stiffness, as changes in wavelength are sometimes visible and these correspond to changes in stiffness (in the absence of boundary effects). For quantitative results, however, reconstruction is used. Typically, due to the time harmonic excitation, dynamic vibrational analysis is used, which models the displacements and stiffness parameters as complex‐valued entities. The equations, in this form, then also capture effects of viscoelasticity. A discrete Fourier transform converts the time‐dependent real displacements, **u**_*r*_(**x**,*t*), into a steady‐state complex‐valued field, **u**(**x**), related by $u_{r}(x,t)=\text{Re}\left[u(x)\exp(i\omega t)\right]$, and reconstruction methods calculate complex values for stiffness, *G*.

The isotropic linear viscoelasticity equations are

(1) $$\rho \omega^{2}u+\nabla \cdot \left[G\left(\nabla u+{\left(\nabla u\right)}^{T}\right)\right]+\nabla p=0$$

(2) $$\nabla \cdot u-\frac{p}{\lambda }=0$$

where **u**, *p*, *G* and λ are complex valued. Additionally, *p* is pressure and *ω* is angular frequency. The density, *ρ*, is usually assumed to be that of water, *ρ*≈1000 kg/m^3^, when modeling tissue. The shear modulus, *G*, is often written as *G*=*G* *′*+*i* *G* *′* *′*, where *G* *′* is the storage modulus and *G* *′* *′* is the loss modulus. Generally, Equation 2 can be inserted into Equation 1, giving

(3) $$\rho \omega^{2}u+\nabla \cdot \left[G\left(\nabla u+{\left(\nabla u\right)}^{T}\right)\right]+\nabla \left(\lambda \nabla \cdot u\right)=0$$

although this is typically only done for compressible materials. Often, tissue is modeled as incompressible and Equation 2 becomes the divergence‐free condition on the displacements,

(4) ∇·u=0

In a typical forward problem, the material parameters are known and **u** and *p* are unknown. Boundary conditions are also necessary. For example, Dirichlet conditions such as $u=\bar{u}$ may be specified, where $\bar{u}$ are known displacements.

The weak (or variational) form of these equations is also noteworthy, as it is the weak form on which the finite‐element method (FEM) operates. The FEM is a popular numerical discretization choice for MRE because, as an integral approach, it reduces the order of differentiation, and thereby may help to reduce noise sensitivity. For example, the incompressible forward problem defined by Equations 1 and 4 has the weak form: find $\left(u,p\right)\in U\times L_{C}^{2}\left(\Omega \right)$ such that, for all $\left(w,q\right)\in U_{0}\times L_{C}^{2}\left(\Omega \right)$,

(5) $$\int_{\Omega }\left(\rho \omega^{2}u\cdot w-\left[G\left(\nabla u+{\left(\nabla u\right)}^{T}\right)\right]:\nabla w+p\;\nabla \cdot w\right)d\Omega +\int_{\Gamma_{2}}\bar{T}\cdot w\;d\Gamma_{2}=0$$

(6) $$\int_{\Omega }q\;\nabla \cdot u\;d\Omega =0$$

with the trial function space $U=\left\{u|u\in H_{C}^{1}\left(\Omega \right),u=\bar{u}\;\text{on}\;\Gamma_{1}\right\}$ and the test function space $U_{0}=\left\{w|w\in H_{C}^{1}\left(\Omega \right),w=0\;\text{on}\;\Gamma_{1}\right\}$. Here, **w** and *q* are test functions, Ω is the spatial domain, Γ_1_ denotes the portion of the boundary of Ω where known displacements, $\bar{u}$, are imposed, and Γ_2_ denotes the portion where known tractions, $\bar{T}$, are used.

However, in MRE an inverse problem is solved, where **u**, *ρ*, and *ω* are known and *G*, λ, and *p* are unknown. Boundary conditions may also need to be reconsidered depending on the inverse formulation. Furthermore, only an estimate of **u** is measured from MR; this is a discrete quantity that contains error (e.g. noise or artifacts), herein denoted ${\bar{u}}_{i}$, where *i* counts over the discrete elements of ${\bar{u}}_{i}$, typically one for each voxel. The goal of reconstruction methods is to solve for *G*, based on the measured ${\bar{u}}_{i}$, and, depending on the method, potentially include solutions for either λ or *p*.

The measured displacement fields, ${\bar{u}}_{i}$, inherently contain noise, as MRI produces images with finite signal‐to‐noise ratio (SNR). A common and straightforward method to reduce noise is to apply a local Gaussian filter directly to the displacement data. Another common approach is local polynomial fitting, also called Savitzky–Golay filtering. From this method, smooth derivatives of the displacement fields can also be computed by using the derivatives of the polynomials calculated during the filtering. These local spatially based filters may have issues near discontinuities and boundaries, but these errors will be localized. Frequency‐based low‐pass filtering is another option, but this may have issues in oddly shaped domains, and, being a global operation, errors may propagate throughout the domain. More advanced denoising techniques have also been applied in MRE^39^. Noise, in part, limits the resolution of MRE for many reconstruction methods, as high‐resolution images would lead to many pixels per wavelength and noise would then dominate many calculations, instead of the curvature of the waves themselves. The other part of this balance is a limit to decreasing the wavelength, as this corresponds to increasing the frequency, which will increase attenuation.

Both compressional and shear waves are present in tissue during MRE and it is the combined displacements from both that are collected by imaging. The assumption of linear viscoelasticity accounts for both types of wave; however, it is the shear modulus, and therefore the shear waves, that elastography is oriented towards. In part, this is due to the shear‐wave speed varying more over different tissue types than the compressional wave speed, but also to the fact that the compressional waves are much faster, which makes balancing considerations like applied frequency and imaging constraints more difficult.^6^ However, since the compressional wave is represented in the imaged displacements, it must somehow be considered during reconstruction. In the equations, the compressional component corresponds to Equation 2 and the *∇* *p* term in Equation 1 or $\nabla \left(\lambda \nabla \cdot u\right)$ in Equation 3.

Amongst reconstruction methods, there are five common approaches for considering the compressional wave: (1) ignore this term, i.e. assume the gradient of pressure is negligible; (2) apply a discretized curl operator to the displacement data and reconstruct using this curl field, assuming it to be divergence‐free; (3) prescribe one of the parameters, most often by recasting Equation 3 in terms of *ν* and *E*, assuming a near‐incompressible value for *ν*, and solving for *E*; (4) apply a high‐pass filter aimed at removing the long‐wavelength compressional wave; and (5) assume incompressibility and solve Equation 1 for *p* in addition to *G*.

There are strengths and drawbacks to each of these approaches. Neglecting the pressure term has been shown to lead to errors in the reconstructed stiffness,^40^ and, while this approach is mentioned as a possibility in several early MRE manuscripts, it is less common in contemporary methods. Applying the curl and attempting to limit hydrostatic effects increases the order of derivatives applied to the displacement field. Since the displacements contain noise and differentiation amplifies noise, this presumably increases the sensitivity of the reconstruction. Using an accurate near‐incompressible value of *ν* typically leads to numerical instability, so usually values in the range 0.49–0.499 are chosen. This causes the errors in the reconstructed λ to be of several orders of magnitude. Reconstructed *G* may be accurate, however there may be errors in regions of strong mode conversion.

When applying a high‐pass filter, it is not always the case that the compressional and shear components are well separated in frequency space. The shape of the domain and areas of mode conversion can lead to a lack of separation. Finally, solving for *p* increases the number of unknowns, thereby demanding more from the available data. The pressure in some cases may also require some regularization. Two works^38^, ^41^ have suggested using specialized virtual fields and FEM test functions, respectively, to remove the compressional component from the equations. This seems to be most similar to including the second unknown (bulk modulus or pressure) in the solution, but could be considered as its own category.

While the preceding discussion has focused entirely on theory assuming harmonic excitation, as this is most common in MRE, some of the methods described in Section 3 assume quasi‐static deformation. Both of these approaches assume a steady state and so techniques from one category may be applicable to the other.

## 3 METHODS

Reconstruction methods for MRE may be categorized in a variety of ways, but probably the largest division regards the two approaches to solving the inverse problem: *iterative* and *direct*. In other literature, iterative methods are sometimes called nonlinear inversion (NLI) methods.

Iterative methods solve a nonlinear constrained minimization problem, that is they attempt to minimize the difference between the measured wave field and simulated wave fields found from solving forward problems. During each iteration, a forward problem is solved using a set of stiffness variables calculated from a previous iteration using minimization update techniques. Upon reaching a minimum, or satisfying some convergence criteria, the current stiffness variables are considered to be the solution to the inverse problem. Iterative methods, therefore, are strongly dependent on model assumptions such as initial stiffness values and boundary conditions, as they only consider displacement fields that satisfy the governing equations. Features in the landscape of the stiffness variable space, such as multiple minima or shallow minima, may lead to a lack of uniqueness, slow convergence, or divergence. Regularization techniques are often employed to mitigate these issues, as well as decreasing the likelihood of inaccurate or non‐physical results. These come with the drawback of additional parameters, on which the results will also depend. Iterative methods are currently computationally expensive, due to the many matrix solutions required for computing both the forward solutions and the stiffness variable updates; however, many potential advances may address this. Despite these concerns, iterative methods are a useful approach, due to their relative independence from data quality and their potential for increased accuracy.

In contrast, direct methods, while having many benefits, are more dependent on data quality. They solve a linear minimization problem, by assuming that the displacements from the measured wave field are sufficiently accurate to be inserted into the governing equations, leaving the linearly dependent stiffness variables (and potentially pressure) as the unknowns available to minimize the system. After computing any required displacement derivatives by standard numerical techniques, the system is solved directly for the unknowns. Typically, the resulting matrix system is overdetermined and so is solved by least‐squares matrix inversion. Due to the single solution, these methods require much less computational effort than iterative methods. Other advantages include fewer method parameters and the guaranteed existence and uniqueness of a solution. The solution itself is, however, more sensitive to data quality and any processing parameters, compared with iterative methods.

### 3.1 Iterative methods

The goal of minimization in iterative methods is to find

(7) $$\min_{\left(\hat{u},p,G\right)}\|\hat{u}-\bar{u}{\|}_{X}$$

 subject to $L(\hat{u},p,G)=0$, where $\hat{u}$ are calculated displacements, $\bar{u}$ are measured displacements, ${\left\|\cdot \right\|}_{X}$ denotes some norm, and the operator, L, is presumably based on the equations of motion presented in Section 2.

The measured displacement data are often used in two ways within iterative methods. The first is to define the optimal configuration of the displacements calculated from the forward problem, as in Equation 7. The second is to specify the boundary conditions for the forward problem. Along with boundary conditions, an initial distribution of stiffness must also be chosen, at which point a forward problem may be solved for displacements via numerical discretization techniques, such as FEM. From here, some optimization algorithm is followed in order to update the stiffness. This typically requires computing changes in the solution due to changes in stiffness, which, in turn, requires solving a similar forward problem for each discrete stiffness parameter. The computed stiffness update is applied and a new forward problem is solved with this updated distribution. This process repeats until some stopping criterion is reached. Common minimization approaches include gradient descent, Gauss–Newton, and Newton's method. In this order, these methods are increasing in the amount of computation required per iteration but decreasing in the number of iterations required in theory. It seems that the most common in MRE reconstruction is Gauss–Newton, as this does not require construction of the second‐order derivatives for Newton's method but offers a more accurate update than gradient descent.

One straightforward and common way to solve Equation 7 is least‐squares, which attempts to minimize the function

(8) $$F(G)=\sum_{i=1}^{N}\sum_{j=1}^{3}{\left({û}_{ij}-{ū}_{ij}\right)}^{*}({û}_{ij}-{ū}_{ij})$$

where **G** is a vector containing the material parameters, *i* counts over voxel locations, *j* counts over the three components of the displacements, and ∗ denotes the complex conjugate. Derivatives of *F* with respect to each material parameter are computed and an update formula is constructed, typically of the form

(9) $$G_{n+1}=G_{n}+\Delta G_{n}$$

where Δ*G*_*n*_ is found by solving (for a Gauss–Newton approach)

(10) $$(J^{T}J)\Delta G_{n}=-J^{T}r$$

Here, **r** is the residual vector and **J** contains the derivatives of *F*.

A common modification to the Gauss–Newton optimization algorithm is Levenberg–Marquardt.^42^ This approach weights the approximate Hessian, **J** ^T^ **J**, towards a gradient descent system by an adaptively updated weighting parameter. This may be thought of as regularization of the approximate Hessian, but, importantly, this strategy does not change the stiffness variable landscape, only the approach towards a minimum. Considering the form presented in Equation 10, the gradient‐descent method simply replaces **J** on the left‐hand side with the identity and typically a multiplier is used to limit the step size. A full Newton's method approach, which would replace **J** ^T^ **J** with the Hessian matrix, has not yet been applied to elastography. Other options include quasi‐Newton methods, such as BFGS, which approximate the Hessian as the iterations proceed.

Regularization of the stiffness parameters is also commonly employed, often by adding some measure of the derivatives of the stiffness to the minimization function, thereby encouraging a smoother result. Tikhonov regularization, for example, involves adding the *L* ^2^ norm of the Laplacian of the stiffness.^43^ These techniques usually require this additional regularization term to include a weighting parameter to weight this portion of the minimization against the norm of the displacement differences. This type of regularization changes the stiffness variable landscape, favoring smoother solutions and ideally improving the likelihood of a clear minimum.

#### 3.1.1 Gradient‐descent methods

**Figure 3:**
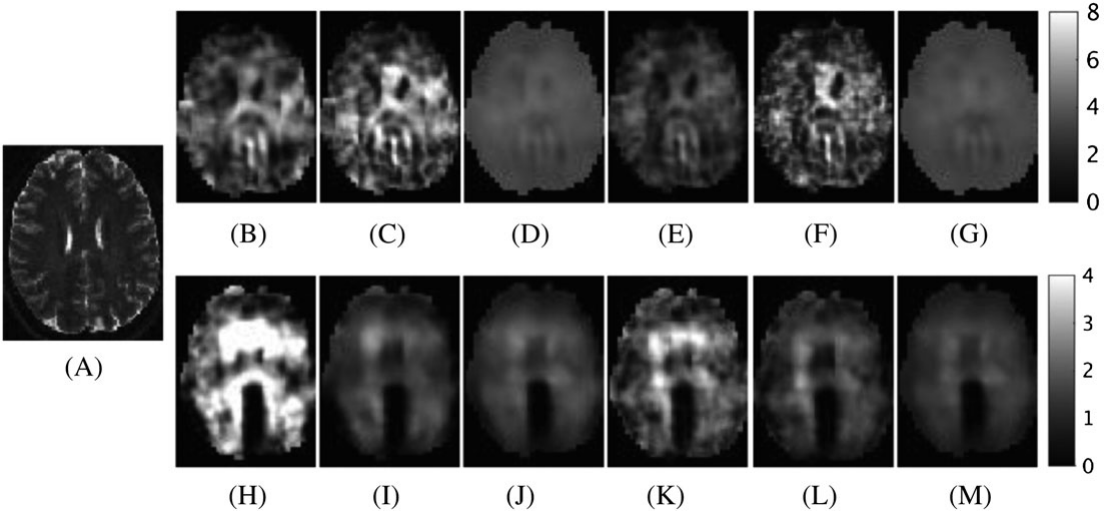
Comparisons of brain MRE data reconstructed using conjugate gradient, quasi‐Newton, and Gauss–Newton methods and compared with A, a T_2_‐weighted image. B–D, G ′ reconstructions are shown using the compressible equations for the three methods in the order previously listed. E–G, G ′ reconstructions are also shown using the incompressible equations. H–M, The corresponding map of G ′ ′. Values are in units of kPa (©2017 IEEE. Reprinted, with permission, from Tan et al^46^

The application of gradient‐descent methods to elastography has been investigated by Oberai et al,^44^, ^45^ in which an adjoint operator is used to modify the linear elasticity equations to realize the descent calculation. This is more efficient than standard gradient‐descent methods, as it requires only two solutions for each iteration. The mixed displacement‐pressure formulation of the static deformation equations are solved via FEM in 2D and the minimization is regularized via a Tikhonov approach. Tan et al^46^ introduced a generalized adjoint method applicable to poroelasticity and also provided comparisons between conjugate‐gradient, quasi‐Newton, and Gauss–Newton methods by computational complexity and by results across various data sets, including phantom and brain (Figure 3).

#### 3.1.2 Subzone

A particularly prominent iterative reconstruction method, and one usually oriented towards harmonic MRE, is the subzone technique, proposed by Van Houten et al.^47^ In later publications, this method was evaluated further^48^ and extended to 3D.^49^, ^50^ This method has typically been implemented with Gauss–Newton, but also with quasi‐Newton methods,^51^ and usually employs Levenberg–Marquardt. This approach uses a FEM discretization of the equations and performs a minimization procedure in many small overlapping domains, or subzones, that span the global domain. Both local and global convergence are usually considered. While specifics depend on the particular implementation, typically some minimization iterations are performed in the subzones and, at certain intervals or after local convergence, the subzone domains are changed and values are exchanged between them. Another round of minimization within subzones is performed and the process repeats until a global convergence criterion is satisfied. In various implementations of this method, either *ν* is set to a near‐incompressible value and *E* is solved for, both *ν* and *E* are solved for, or both λ and *G* are solved for.

Many updates and extensions to this method have been published. A parallelized 3D implementation was described^52^ and parameter choices for this implementation were analyzed later.^53^ Density was added as an unknown parameter,^54^ a Rayleigh‐damped model has been considered,^55^ and poroelasticity has been investigated.^56^, ^57^ A multiresolution approach was developed^58^ and soft prior regularization was presented, which predefines areas of homogeneous material parameters.^51^ The method has been used in many studies, including those on SNR,^59^ frequency and direction dependence in phantoms,^60^ and various other *in vivo* and *ex vivo* data (Table 2). Doyley et al^52^ reported computation times of 1.5–4.5 hours by varying the subzone size (twelve 2.5‐GHz Xenon IV compute nodes running Linux with problem sizes greater than 10^4^ FEM nodes). Perreard et al^60^ reported approximately 5 hour run times (eight AMD opteron processors running 2.5×10^5^ element meshes with 100 global iterations.)

#### 3.1.3 Gauss–Newton methods

Many other reconstructions based on Gauss–Newton iterations have been published, in addition to the subzone technique. Kallel and Bertrand^61^ solved the static elasticity equations in 2D with Tikhonov regularization and near‐incompressible values of *ν*. Later, this approach was modified to utilize total variational regularization.^62^ Doyley et al^63^ and Miga^64^ followed similar approaches and used Levenburg–Marquardt. While the previous studies were oriented towards US, Khalil et al^18^ followed this approach for OCT with Tikhonov regularization.

Eskandari et al^65^ solved the time‐harmonic equations in a similar fashion using Levenburg–Marquardt. This implementation was later used in a study on bandpass sampling^66^ and was extended to employ mesh adaptation.^67^ Honarvar et al^68^ solved the mixed displacement‐pressure and time‐harmonic form using Gauss–Newton and Levenburg–Marquardt. This implementation was also used for comparison with heterogeneous direct methods.^68^

#### 3.1.4 Traveling‐wave expansion

Baghani et al^69^ introduced the traveling‐wave expansion (TWE) method for MRE. This method fits fundamental solutions of the Helmholtz equation to the measured displacement data and also assumes local homogeneity of stiffness. The solution chosen for fitting is a summation over a finite set of directions of complex exponential functions with a constant (and complex) wave number. The method works voxel by voxel and, for any wave number, a linear minimization is performed to calculate the unknown parameters in the solution function, thereby fitting the solution to some local subset of the data. The method is categorized as iterative here, because it performs an additional minimization over possible complex wave numbers, which is achieved by a Newton‐type iteration, so, at each voxel, the method computes the wave number that minimizes the error between the data and a local fit of the data. The wave number for the compressional component is prescribed. Although an iterative solution is required at every voxel, the iterations only involve two parameters and, when fitting, the system sizes are small due to the local constructions. This keeps the computation time low and times of less than one minute are reported for image sizes of the order of 10^4^ voxels in phantom studies,^69^ although no CPU information appears to be given.

#### 3.1.5 Other

Samani et al^70^ introduced a method that uses the linear elasticity equations themselves as an update formula for iterating towards the solution. This method uses FEM and considers the quasi‐static 3D equations with *ν* set to a near‐incompressible value. A study published later used and extended this method.^71^ A genetic algorithm approach was presented by Zhang et al,^72^ which also uses FEM and a near‐incompressible *ν*. Fu et al^73^ considered local stiffness homogeneity within an iterative approach. This method performs a minimization procedure at each voxel by considering increased and decreased stiffness and finding the midpoint. This method assumes 2D and a near‐incompressible *ν*. As with the TWE approach, the homogeneity assumption significantly reduces computational time.

### 3.2 Direct methods

Direct methods take advantage of the abundance of data acquired in MRE to solve directly for the stiffness. This leads to a simpler approach than iterative methods, usually requiring fewer parameters and much less computational effort. If stiffness is allowed to vary spatially, then regularization is sometimes still required to stabilize the solution. These heterogeneous direct methods typically solve over the entirety (or large portions) of the domain and attempt to capture the true heterogeneous nature of the stiffness. In contrast, local direct methods assume local homogeneity of stiffness and perform independent solutions on many small subdomains that combine to span the full domain.

While the accuracy of local methods will suffer in regions with significant stiffness heterogeneity, the homogeneity assumption simplifies and stabilizes the methods as well as decreasing computational expense. In the simplest case of local direct inversion, and where the compressional component is assumed to have been removed, the stiffness at voxel *i* could be found by least squares:

(11) $$G_{i}=\frac{-\rho \omega {\left(\bar{\Delta }u\right)}_{i}^{T}{\bar{u}}_{i}}{{\left(\bar{\Delta }u\right)}_{i}^{T}{\left(\bar{\Delta }u\right)}_{i}}$$

where ${\bar{u}}_{i}$ is the displacement data vector and ${\left(\bar{\Delta }u\right)}_{i}$ is some approximation of the Laplacian of the data at voxel *i*.

#### 3.2.1 Heterogeneous direct methods

Heterogeneous direct methods consider stiffness to vary spatially and invert the governing equations directly with the proper derivatives affecting the stiffness. These methods are less computationally expensive than iterative methods, as only a single solution is required, but are more expensive than local direct methods, as the matrix system is much larger. Allowing for stiffness heterogeneity may lead to more instability and sensitivity in the method, and therefore these methods sometimes require regularization. As with iterative methods, a common regularization approach is Tikhonov, where, again, a measure of the Laplacian of the stiffness is added to the minimization. If pressure is additionally solved for, then this may also be regularized.

Several methods consider 2D quasi‐static elasticity with near‐incompressible values of *ν* and discretization via FEM.^74^, ^75^, ^76^ Eskandari et al^76^ suggested a quadratic programming technique, which constrains stiffness values to physically reasonable ranges. An adjoint weighted equation formulation was presented and demonstrated in multiple instances such as compressibility and anisotropy.^77^, ^78^ Zhang et al^79^ extended this approach to the time‐harmonic case in 2D with a mixed formulation and total variation regularization. Effects of noise on this method were presented later.^80^

Park and Maniatty^40^ solved the mixed displacement‐pressure form of the harmonic linear elasticity equations. In this approach, FEM is used with Tikhonov‐type regularization applied to the pressure, whereas the stiffness is not regularized. The measured displacement data are filtered by solving for a new displacement field that minimizes three terms: the difference compared with the measured displacements, the divergence of the field, and the Laplacian of the field. Between the pressure regularization and displacement adjustment, at least five regularization parameters are introduced. In the FEM solution, all equations associated with boundary nodes are removed, which removes the unknown boundary terms in Equation 5. This technique was also used in Eskandari et al^76^ and the following heterogeneous methods.

Honarvar et al^81^ suggested a similar process to reconstruction to Park and Maniatty,^40^ but used sparsity regularization instead of Tikhonov for both pressure and stiffness. In Honarvar et al, the sparsity regularization is performed using the discrete cosine transform and masking in frequency space to limit the stiffness and pressure distributions to lower frequency contributions, effectively smoothing the result. As opposed to Tikhonov, sparsity reduces the size of the matrix system, but also globalizes the solution, filling the otherwise sparse and banded matrix. Removing these properties of the system should lead to a significant increase in computation time, unless only very few frequencies are included. On the other hand, filling the matrix could help to stabilize the solution.

**Figure 4:**
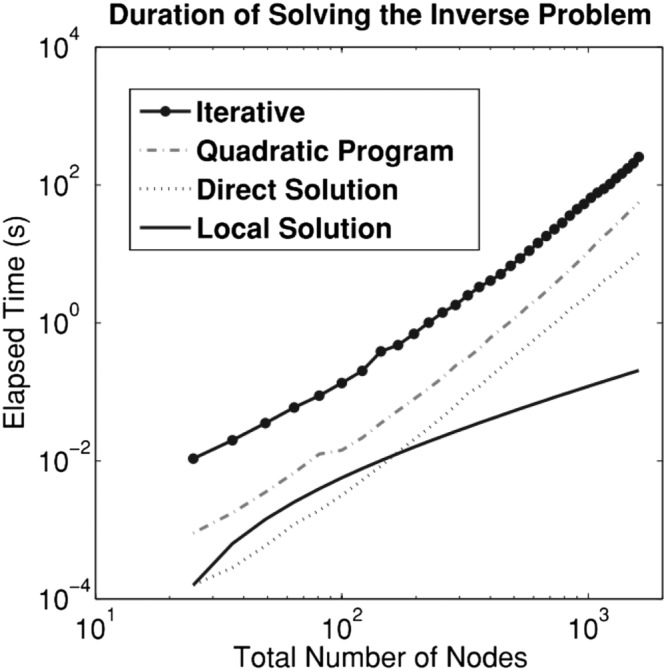
Plot comparing the computational cost of various methods considering 2D data (measured on a quad‐core CPU with two 2.82‐GHz processors): a single iteration of an iterative method,^65^ a heterogeneous direct method, a local direct method, and the quadratic programming technique outlined in Eskandari et al.^76^ (©IOP Publishing. Reproduced with permission. All rights reserved. Eskandari et al. 2011)

A similar method was later introduced in which the curl operator is applied to the equations to remove the pressure term.^82^ In the finite‐element process, integration by parts is applied for an additional time to what is standard in order to remove the extra derivative introduced by the curl. Then, in order to remove the boundary conditions, special basis functions are utilized. Similar results were reported between the mixed and curl forms, although, since removing the pressure term reduces the number of unknowns, the curl form is computationally faster.^82^ The mixed form was later compared with an iterative method.^68^ Computation times of 15 minutes were reported for the curl form for 3D data and 35 minutes for the mixed form^82^ (computed on 15×15×15 overlapping subdomains of a 140×46×30 ROI with a MATLAB implementation, but CPU information was not reported). In Fovargue et al,^38^ computation times of approximately 50–90 seconds were reported for data sets with approximately 24 000–33 000 voxels for a mixed heterogeneous implementation with Tikhonov regularization (MATLAB with an Intel Xeon 4‐core, 8‐thread, 3.6 GHz processor). In Eskandari et al,^76^ compute times for the 2D heterogeneous direct method were compared with those of an iterative method^65^ and local method for varying image sizes (Figure 4).

#### 3.2.2 Local frequency estimation

Manduca et al^83^ presented a local direct method called the local frequency estimation (LFE) method, which is based on particular frequency banks and filtering described in Knutsson et al.^84^ The stiffness is then calculated from the frequency estimate by assuming homogeneity and no attenuation (meaning only the real part of the complex stiffness modulus is provided). Although the compressional component is not accounted for directly, its effect may be minimized by limiting the frequency range during filtering. Braun et al^85^ investigated the use of Gauss filters in place of the lognormal quadrature filters used previously^83^ and principal frequency estimation was proposed by McGee et al.^86^ LFE has also been used to investigate the effect of off‐frequency sampling,^87^ as well as in many *ex vivo* and *in vivo* animal and human studies (Table 2).

#### 3.2.3 Direct inversion

Direct inversion methods (sometimes denoted DI) perform a pixel‐wise inversion of the homogeneous time‐harmonic linear viscoelasticity equations. This was first suggested in Romano et al^88^, ^89^ using a variational approach and Oliphant et al^90^, ^91^ using polynomial fitting to compute the necessary derivatives. In these first implementations, the compressional component is sometimes ignored. Manduca et al, proposed high‐pass filtering of the wave data in order to limit the low‐frequency compressional component.^92^, ^93^ Manduca et al^93^ also presented spatio‐temporal directional filtering as a pre‐processing step and compared results from direct inversion and LFE algorithms. Sinkus et al^94^ followed a similar procedure to find tensor components of stiffness with a near‐incompressible value for *ν*. Another common approach among direct inversion implementations is to apply a discrete curl operator to the displacement data and reconstruct using the curl field.^92^, ^95^

Okamoto et al^96^ proposed a finite‐difference‐based direct inversion using total least squares, which may provide more accuracy when there is uncertainty in the independent variable. This work also compared MRE with dynamic shear testing in soft gels. Connesson et al^41^ implemented a virtual fields method approach, wherein the virtual field is constructed to remove the bulk term and reduce dependence on noise. Fovargue et al.^38^ proposed a FEM‐based inversion that uses a specific basis for the FEM test functions to remove the pressure term. Computation times for local direct methods are also supplied in Fovargue et al,^38^ where times ranging from 3–9 seconds were reported (MATLAB with an Intel Xeon 4‐core, 8‐thread, 3.6‐GHz processor).

Direct inversion methods have been used extensively in both research and clinical studies. McLaughlin et al^97^ used this approach to investigate filtering. Papazoglou et al^98^ investigated factors such as SNR and actuator orientation on the resulting elastogram and Riek et al^99^ investigated the frequency dependence of stiffness. Table 2 contains many examples of DI methods applied to human and animal studies, where, to provide some division, curl‐based approaches are listed separately from non‐curl‐based approaches.

#### 3.2.4 Multifrequency direct inversion

**Figure 5:**
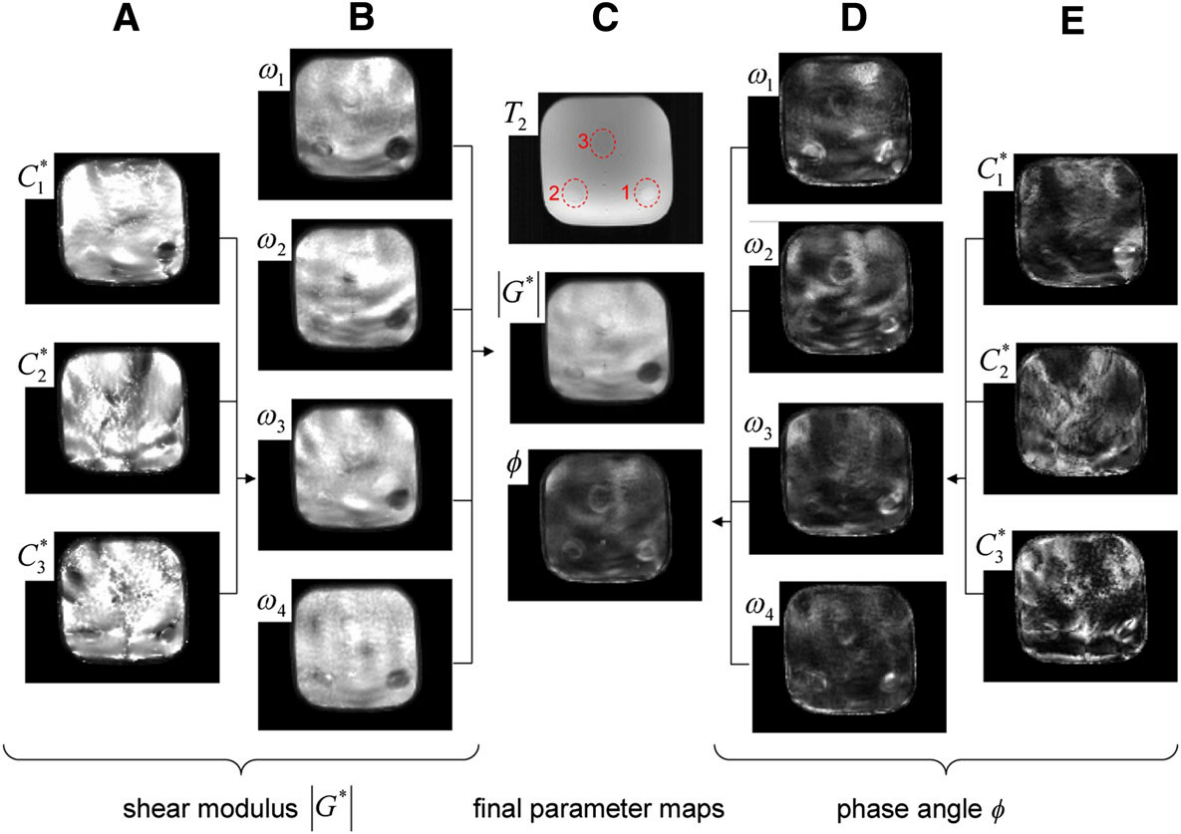
Diagram showing the process used in the MDEV inversion. The curl components (columns A and E) are used to find the magnitude and phase of G for each multifrequency data set (columns B and D), which is averaged in column C by comparison with a T_2_‐weighted image (©2013 Wiley Periodicals, Inc. Reproduced, with permission, from Hirsch et al^101^

Combining multiple‐wave data sets corresponding to the application of different vibrational frequencies is one potential avenue for increasing the stability and reliability of MRE, by essentially increasing the amount of data per unknown. This comes with the disadvantages of needing to account for the frequency dependence of stiffness and potentially longer scan times. Papazoglou et al^100^ proposed a local direct inversion method using high‐pass filtering to limit the compressional component and utilizing multiple data sets representing different frequencies. The stiffness is computed in accordance with a springpot power law after first computing the exponent parameter. Hirsch et al^101^ proposed a similar approach called multifrequency dual elastovisco (MDEV) inversion. The phase and magnitude of the complex shear modulus are computed separately and using averages over the multifrequency data sets. This method also computes and uses the curl of the wave data. A diagram illustrating the process is shown in Figure 5. MDEV is also used as the inversion algorithm in the elastography pipeline presented by Barnhill et al,^39^ as well as some studies on human data (Table 2). Tzschatzsch et al^102^ introduced a 2D multifrequency approach called k‐MDEV, which uses filters in frequency space to construct a set of plane‐wave approximations in multiple directions (these filters also work to limit the compressional component). Estimates of the stiffness and attenuation are found from plane‐wave assumptions and by averaging over all frequencies and plane‐wave directions. Multifrequency inversion is also applicable to other reconstruction methods, not only local direct inversion, and this is an area of active research.

## 4 DISCUSSION AND CONCLUSION

The methods presented in Section 3 are also summarized and categorized in Table 1, based on approaches to the inverse solution, local homogeneity assumption, and compressional component of the wave data. Some of the methods have been successfully applied to *ex vivo* and *in vivo* human and animal data. A summary of a selection of these studies is provided in Table 2. MRE has covered many organs, each with their own challenges as well as specific disease and treatment response areas. The liver fibrosis and brain sections of Table 2, for example, contain many studies, as these have shown much promise with MRE. More challenging areas include heart and skeletal muscle. Heart data often do not contain enough pixels to apply traditional reconstruction techniques, so, in addition to the one study listed in Table 2, other studies have measured relative changes in stiffness by examining changes in shear‐wave amplitude^103^, ^104^, ^105^ and by utilizing a spherical shell model.^106^ Due to the strong anisotropy present in muscle, some methods, after carefully considering the wave propagation direction, apply damped sine‐wave fits^107^ or simple peak‐to‐trough measurements.^108^, ^109^, ^110^, ^111^

**Table 1:** A summary of many of the reconstruction methods mentioned in this review. Here, they are presented in chronological order and categorized in terms of their approach to the inverse problem, whether they assume local stiffness homogeneity, and their consideration of the compressional component of the wave data. For the latter category, the following symbols are used: IG – ignore; ∇× – compute the curl of the wave data; NI – prescribe a near‐incompressible value; P – additionally solve for pressure in the mixed displacement‐pressure formulation; HPF – apply a high‐pass filter to the data; Gλ – solve for both the shear modulus, G, and the first Lamé parameter, λ

Method	Inverse problem approach	Local stiffness homogeneity	Compressional component
Kallel et al^61^	Iterative	No	NI
Manduca et al^83^	Direct	Yes	IG
Romano et al^88^	Direct	Yes	IG
Oliphant et al^90^	Direct	Yes	IG
Van Houten et al^47^	Iterative	No	NI
Doyley et al^63^	Iterative	No	NI
Fu et al^73^	Iterative	Yes	NI
Sinkus et al^94^	Direct	Yes	NI
Manduca et al^92^	Direct	Yes	HPF
Samani et al^70^	Iterative	No	NI
Van Houten et al^49^	Iterative	No	NI
Miga et al^64^	Iterative	No	NI
Oberai et al^44^	Iterative	No	P
Zhu et al^74^	Direct	No	NI
Doyley et al^52^	Iterative	No	Gλ
Sinkus et al^95^	Direct	Yes	*∇*×
Park and Maniatty^40^	Direct	No	P
Eskandari et al^65^	Iterative	No	NI
Guo et al^75^	Direct	No	NI
Baghani et al^69^	Iterative	Yes	NI
Eskandari et al^76^	Direct	No	NI
Okamoto et al^96^	Direct	Yes	IG
Honarvar et al^81^	Direct	No	P
Papazoglou et al^100^	Direct	Yes	HPF
Zhang et al^79^	Direct	No	P
Honarvar et al^82^	Direct	No	*∇*×
McGarry et al^51^	Iterative	No	NI
Hirsch et al^101^	Direct	Yes	*∇*×
Connesson et al^41^	Direct	Yes	Gλ
Honarvar et al^68^	Iterative	No	P
Tzschätzsch et al^102^	Direct	Yes	HPF
Tan et al^46^	Iterative	No	P
Fovargue et al^38^	Direct	Yes	P

**Table 2:** Many *in vivo* and *ex vivo* human and animal studies using MRE have been published and a selection are listed in this table. The location in the table indicates the area of the study (row) and the type of reconstruction used (column). The first portion of the table includes studies on particular disease and treatment areas, while the second portion covers specific organs. The highlighted methods are as follows: LFE – local frequency estimation method; DI – direction inversion methods using high‐pass filtering or prescribed near‐incompressible values to address the compressional wave; CDI – direction inversion methods using the curl to address the compressional wave; MDEV – multifrequency dual elastovisco inversion method (Section 3.2.4); Subzone – iterative method as described in Section 3.1.2. The last category (Other) includes two studies using a global direct method with sparsity regularization^120^, ^176^ and one using TWE^174^

Area	LFE	DI	CDI	MDEV	Subzone	Other
Brain cancer			112	113		
Breast cancer	114, 115	94	28, 116			
Liver cancer	29		117			
Prostate cancer	118		119			120
Liver fibrosis	24, 121, 122, 123, 124, 125		25			
Alzheimer's			31		30	
Parkinson's				32		
Multiple sclerosis		33, 126	34	126	127	
Ablation	128, 129					
Liver response	130, 131					
Tumor response		132	133			
Brain	134	135, 136, 137, 138, 139, 140, 141	112, 142, 143, 144, 145	146, 147	148, 149, 150, 151	
Breast	152					
Heart	153		154			
Kidney	155, 156, 157		158	159		
Liver	160	136				
Lung	161, 162, 163, 164	165				
Muscle		166, 167	168, 169, 170, 171			
Pancreas	172		173			
Prostate	174	175				174, 176

Although the iterative subzone, TWE, and global direct with sparsity regularization methods have been used in a few studies, local direct methods seem to dominate Table 2. Also, these methods, particularly LFE, have been implemented more often in software made available to clinicians and other researchers, such as the LFE method in the MRE Wave software (Mayo Clinic, Rochester, MN, USA) and Resoundant software (Rochester, MN, USA), as well as the MDEV, k‐MDEV, and LFE methods on a publicly accessible website.^177^ Several possible reasons for this disparity include low computation time, ease of use, and guaranteed solutions.

Local direct methods are by far the least computationally expensive; however, few publications on these methods^38^, ^76^ provide computation time estimates. This is presumably because they are too short to be of concern. Even for larger 3D data sets, the reconstruction time is measured in seconds (this is not including steps such as phase unwrapping; only stiffness reconstruction from properly processed displacement data). In contrast, computation times of heterogeneous direct methods are typically measured in minutes. These methods sometimes rely on regularization parameters being optimized, so may need to be run multiple times. This may also be true of the even more expensive iterative methods. The computation times of these methods, however, may vary considerably, as they depend strongly on implementation, domain size, and parameters. Times measured in hours have been reported, but with increasing computing power, domain‐size reduction, and algorithmic advances, those times may be significantly reduced. Overall, few direct comparisons of computational time between methods have been reported and the comparisons made here should be taken as mostly anecdotal, as the reported times span years and are performed on different implementations with different CPU specifications.

Furthermore, local direct methods are usually not dependent on parameters, besides those controlling the initial smoothing of the wave field. While some options may be available to researchers, these methods are typically able to be used out of the box. Methods that consider stiffness heterogeneity, whether direct or iterative, usually require regularization terms to restrain the stiffness. These terms are weighted against the original equations, but the weights of these parameters are difficult to know *a priori* and can affect the resulting elastogram. Iterative methods, being more complicated, are additionally dependent on parameters other than stiffness regularization, such as subdomain size. While the parameter governing the Levenberg–Marquardt procedure is updated during the iterations, the initial choice may still have an effect. Results and comparisons from iterative and heterogeneous methods can therefore be harder to justify if significant parameter adjustment has occurred.

Reconstruction methods are sensitive to inconsistencies in the wave data, such as noise, but also imaging artifacts or other wave behavior that deviates from material assumptions. It is the regularization mentioned above that decreases sensitivity to these phenomena and allows methods to attain a level of well‐posedness. Local direct methods again excel here. This is due to both the homogeneity assumption, which increases stability and robustness, and the direct approach, which guarantees a unique solution.

Although local direct methods dominate the studies in Table 2, there has been much research devoted to other approaches, as seen in Table 1. This is presumably because of the potential for increased accuracy from heterogeneous and iterative methods. Heterogeneous approaches correctly capture changes in stiffness not only at large discontinuities but also in continuously varying regions. Local direct methods are inaccurate in these regions, to varying degrees, and changes in one component of the complex shear modulus can lead to inaccuracies in both. Furthermore, local methods are not guaranteed to predict an average of the underlying varying stiffness, but instead may calculate incorrect values, which is particularly evident near discontinuities. Iterative methods, additionally, are less sensitive to noise in the data, at least in theory. This also leads to increased accuracy, as noise tends to decrease computed stiffness in direct methods, because noise is seen as high‐frequency waves, which would signal a lower underlying stiffness. Varying data quality is common too, as attenuation of the waves leads to decreasing SNR as distance from the transducer increases.

If iterative and heterogeneous methods can increase ease of use and robustness, they may begin to compete with local direct methods in terms of clinical viability. This may occur through approaches for quality assessment and confidence estimates or through some automatic adjustment of parameters. Perhaps too often, new reconstruction methods, or updates to existing ones, are validated only on *in silico* and phantom wave data, instead of being tested on more difficult anatomical data. Of course, these broader comparisons require well‐established stiffness results or publicly available MRE data sets. Another option is the development of *in silico* data sets that properly capture the added difficulty inherent in anatomical data.

MRE reconstruction may also be extended by considering other material assumptions. Anisotropy is a common feature of tissue and so has been included in some reconstructions.^170^, ^171^, ^178^, ^179^, ^180^, ^181^ Associated studies include investigations into the validity of anisotropic material assumptions,^182^ results of applying isotropic reconstructions to anisotropic data,^60^, ^150^ experimental requirements,^183^ and hardware to produce adequate wave data.^184^ Poroelastic material assumptions have also been studied in MRE^46^, ^56^, ^57^ as well as US elastography.^185^ Nonlinear, usually neo‐Hookean, constitutive laws have been investigated in the context of static elastography.^186^, ^187^, ^188^, ^189^

Many research directions within MRE are progressing as it continues to strengthen as a diagnostic method. It is becoming more widely used in tangential research and in the clinic through MRE packages available for MR systems. Success has been shown in staging liver fibrosis and potentially in stratification of tumor malignancy and various brain diseases. As precision and accuracy improve, it has the additional potential to replace invasive biopsy procedures for diagnoses. Stiffness reconstruction algorithms have played a vital role in positioning MRE at the forefront of medical imaging research through advances in robustness, accuracy, ease of use, and computational cost. Further improvements in these areas can allow MRE usage to become more widespread by increasing reliability and confidence in the approach.

## References

[1] Yeh WC , Li PC , Jeng YM , et al. Elastic modulus measurements of human liver and correlation with pathology. Ultrasound Med Biol. 2002;28:467‐474. 12049960 10.1016/s0301-5629(02)00489-1

[2] Bataller R , Brenner D . Liver fibrosis. J Clin Invest. 2005;115:209‐218. 15690074 10.1172/JCI24282 PMC546435

[3] Krouskop TA , Wheeler TM , Kallel F , Garra BS , Timothy H . Elastic moduli of breast and prostate tissues under compression. Ultrason Imaging. 1998;20:260‐274. 10197347 10.1177/016173469802000403

[4] Paszek MJ , Zahir N , Johnson KR , et al. Tensional homeostasis and the malignant phenotype. Cancer Cell. 2005;8:241‐254. 16169468 10.1016/j.ccr.2005.08.010

[5] Greenleaf JF , Fatemi M , Insana M . Selected methods for imaging elastic properties of biological tissues. Annu Rev Biomed Eng. 2003;5:57‐78. 12704084 10.1146/annurev.bioeng.5.040202.121623

[6] Glaser KJ , Manduca A , Ehman RL . Review of MR elastography applications and recent developments. J Magn Reson Imaging. 2012;36:757‐774. 22987755 10.1002/jmri.23597 PMC3462370

[7] Mariappan YK , Glaser KJ , Ehman RL . Magnetic resonance elastography: A review. Clin Anat. 2010;23:497‐511. 20544947 10.1002/ca.21006 PMC3066083

[8] Krouskop T , Dougherty D , Vinson F . A pulsed Doppler ultrasonic system for making noninvasive measurements of the mechanical properties of soft tissue. J Rehabil Res Dev. 1987;24:1‐8. 3295197

[9] Lerner RM , Huang SR , Parker KJ . “Sonoelasticity" images derived from ultrasound signals in mechanically vibrated tissues. Ultrasound Med Biol. 1990;16:231‐239. 1694603 10.1016/0301-5629(90)90002-t

[10] Yamakoshi Y , Sato J , Sato T . Ultrasonic imaging of internal vibration of soft tissue under forced vibration. IEEE Trans Ultrason Ferroelectr Freq Control. 1990;37:45‐53. 18285015 10.1109/58.46969

[11] Ophir J , Cespedes I , Ponnekanti H , Yazdi Y , Li X . Elastography: A quantitative method for imaging the elasticity of biological tissues. Ultrason Imaging. 1991;13:111‐134. 1858217 10.1177/016173469101300201

[12] Parker KJ , Doyley MM , Rubens DJ . Imaging the elastic properties of tissue: The 20 year perspective. Phys Med Biol. 2011;56:R1‐R29. 21119234 10.1088/0031-9155/56/1/R01

[13] Lewa CJ , Certaines JD . MR imaging of viscoelastic properties. J Magn Reson Imaging. 1995;5:242‐244. 7766988 10.1002/jmri.1880050221

[14] Muthupillai R , Lomas D , Rossman P , Greenleaf JF , Manduca A , Ehman R . Magnetic resonance elastography by direct visualization of propagating acoustic strain waves. Science. 1995;269:1854‐1857. 7569924 10.1126/science.7569924

[15] Plewes D , Betty I , Urchuk S , Soutar I . Visualizing tissue compliance with MR imaging. J Magn Reson Imaging. 1995;5:733‐738. 8748495 10.1002/jmri.1880050620

[16] Sarvazyan A , Hall TJ , Urban MW , Fatemi M , Aglyamov SR , Garra BS . An overview of elastography – an emerging branch of medical imaging. Curr Med Imaging Rev. 2011;7:255‐282. 22308105 10.2174/157340511798038684 PMC3269947

[17] Schmitt JM . OCT elastography: Imaging microscopic deformation and strain of tissue. Opt Express. 1998;3:199‐211. 19384362 10.1364/oe.3.000199

[18] Khalil AS , Chan RC , Chau AH , Bouma BE , Kaazempur MMR . Tissue elasticity estimation with optical coherence elastography: Toward mechanical characterization of in vivo soft tissue. Ann Biomed Eng. 2005;33:1631‐1639. 16341928 10.1007/s10439-005-6766-3

[19] Kennedy BF , Liang X , Adie SG , et al. In vivo three‐dimensional optical coherence elastography. Opt Express. 2011;19:6623‐6634. 21451690 10.1364/OE.19.006623 PMC3308196

[20] Sun C , Standish B , Yang VX . Optical coherence elastography: Current status and future applications. J Biomed Opt. 2011;16:1‐12. 043001. 10.1117/1.3560294 21529067

[21] Kennedy BF , Kennedy KM , Sampson DD . A review of optical coherence elastography: Fundamentals, techniques and prospects. IEEE J Sel Top Quantum Electron. 2014;20:272‐288.

[22] Kim GW , Han BH , Cho MH , Lee SY . X‐ray elastography: A feasibility study In: 2009 Annual International Conference of the IEEE Engineering in Medicine and Biology Society, EMBC. Minneapolis, MN, USA: IEEE; 2009:3513‐3516. 10.1109/IEMBS.2009.5334527 19964803

[23] Egorov V , Sarvazyan AP . Mechanical imaging of the breast. IEEE Trans Med Imaging. 2008;27:1275‐1287. 18753043 10.1109/TMI.2008.922192 PMC2581459

[24] Yin M , Talwalkar JA , Glaser KJ , et al. Assessment of hepatic fibrosis with magnetic resonance elastography. Clin Gastroenterol Hepatol. 2007;5:1207‐1213. 17916548 10.1016/j.cgh.2007.06.012 PMC2276978

[25] Huwart L , Sempoux C , Vicaut E , et al. Magnetic resonance elastography for the noninvasive staging of liver fibrosis. Gastroenterol. 2008;135:32‐40. 10.1053/j.gastro.2008.03.076 18471441

[26] Bonekamp S , Kamel I , Solga S , Clark J . Can imaging modalities diagnose and stage hepatic fibrosis and cirrhosis accurately? J Hepatol. 2009;50:17‐35. 19022517 10.1016/j.jhep.2008.10.016

[27] Venkatesh SK , Yin M , Ehman RL . Magnetic resonance elastography of liver: Technique, analysis, and clinical applications. J Magn Reson Imaging. 2013;37:544‐555. 23423795 10.1002/jmri.23731 PMC3579218

[28] Sinkus R , Siegmann K , Xydeas T , Tanter M , Claussen C , Fink M . MR elastography of breast lesions: Understanding the solid/liquid duality can improve the specificity of contrast‐enhanced MR mammography. Magn Reson Med. 2007;58:1135‐1144. 17969009 10.1002/mrm.21404

[29] Venkatesh SK , Yin M , Glockner JF , et al. MR elastography of liver tumors: Preliminary results. AJR Am J Roentgenol. 2008;190:1534‐1540. 18492904 10.2214/AJR.07.3123 PMC2894569

[30] Pattison A , Lollis S , Perrinez P , et al. Time‐harmonic magnetic resonance elastography of the normal feline brain. J Biomech. 2010;43:2747‐2752. 20655045 10.1016/j.jbiomech.2010.06.008 PMC2963725

[31] Murphy MC , Huston CR , Glaser KJ , Manduca A , Felmlee JP , Ehman RL . Decreased brain stiffness in Alzheimer's disease determined by magnetic resonance elastography. J Magn Reson Imaging. 2011;34:494‐498. 21751286 10.1002/jmri.22707 PMC3217096

[32] Lipp A , Trbojevic R , Paul F , et al. Cerebral magnetic resonance elastography in supranuclear palsy and idiopathic Parkinson's disease. Neuroimage Clin. 2013;3:381‐387. 24273721 10.1016/j.nicl.2013.09.006 PMC3814959

[33] Wuerfel J , Paul F , Beierbach B , et al. MR‐elastography reveals degradation of tissue integrity in multiple sclerosis. Neuroimage. 2010;49:2520‐2525. 19539039 10.1016/j.neuroimage.2009.06.018

[34] Schregel K , Tysiak EW , Garteiser P , et al. Demyelination reduces brain parenchymal stiffness quantified in vivo by magnetic resonance elastography. Proc Natl Acad Sci USA. 2012;109:6650‐6655. 22492966 10.1073/pnas.1200151109 PMC3340071

[35] Chan HW , Pressler R , Uff C , et al. A novel technique of detecting MRI‐negative lesion in focal symptomatic epilepsy: Intraoperative shearwave elastography. Epilepsia. 2014;55(4):e30‐e33. 24588306 10.1111/epi.12562

[36] Chauvet D , Imbault M , Capelle L , et al. In vivo measurement of brain tumor elasticity using intraoperative shear wave elastography. Ultraschall in der Med–Eur J Ultrasound. 2016;37:584‐590. 10.1055/s-0034-1399152 25876221

[37] Doyley M . Model‐based elastography: A survey of approaches to the inverse elasticity problem. Phys Med Biol. 2012;57:R35‐R73. 22222839 10.1088/0031-9155/57/3/R35 PMC3374584

[38] Fovargue D , Kozerke S , Sinkus R , Nordsletten D . Robust MR elastography stiffness quantification using a localized divergence free finite element reconstruction. Med Image Anal. 2018;44:126‐142. 29247876 10.1016/j.media.2017.12.005

[39] Barnhill E , Hollis L , Sack I , et al. Nonlinear multiscale regularisation in MR elastography: Towards fine feature mapping. Med Image Anal. 2017;35:133‐145. 27376240 10.1016/j.media.2016.05.012

[40] Park E , Maniatty AM . Shear modulus reconstruction in dynamic elastography: Time harmonic case. Phys Med Biol. 2006;51:3697‐3721. 16861775 10.1088/0031-9155/51/15/007

[41] Connesson N , Clayton E , Bayly P , Pierron F . Extension of the optimised virtual fields method to estimate viscoelastic material parameters from 3D dynamic displacement fields. Strain. 2015;51:110‐134. 26146416 10.1111/str.12126 PMC4486339

[42] Marquardt DW . An algorithm for least‐squares estimation of nonlinear parameters. J Soc Ind Appl Math. 1963;11:431‐441.

[43] Tikhonov AN , Goncharsky AV , Stepanov VV , Yagola AG . Numerical Methods for the Solution of Ill‐Posed Problems. Netherlands: Springer; 1995.

[44] Oberai AA , Gokhale NH , Feijóo GR . Solution of inverse problems in elasticity imaging using the adjoint method. Inverse Probl. 2003;19:297‐313.

[45] Oberai AA , Gokhale NH , Doyley MM , Bamber JC . Evaluation of the adjoint equation based algorithm for elasticity imaging. Phys Med Biol. 2004;49:2955‐2974. 15285258 10.1088/0031-9155/49/13/013

[46] Tan L , McGarry MD , Houten EEV , et al. Gradient‐based optimization for poroelastic and viscoelastic MR elastography. IEEE Trans Med Imaging. 2017;36:236‐250. 27608454 10.1109/TMI.2016.2604568 PMC5256858

[47] Van Houten E , Paulsen K , Miga M , Kennedy F , Weaver J . An overlapping subzone technique for MR‐based elastic property reconstruction. Magn Reson Med. 1999;42:779‐786. 10502768 10.1002/(sici)1522-2594(199910)42:4<779::aid-mrm21>3.0.co;2-z

[48] Van Houten EE , Weaver JB , Miga MI , Kennedy FE , Paulsen KD . Elasticity reconstruction from experimental MR displacement data: Initial experience with an overlapping subzone finite element inversion process. Med Phys. 2000;27:101‐107. 10659743 10.1118/1.598861

[49] Van Houten EE , Miga MI , Weaver JB , Kennedy FE , Paulsen KD . Three‐dimensional subzone‐based reconstruction algorithm for MR elastography. Magn Reson Med. 2001;45:827‐837. 11323809 10.1002/mrm.1111

[50] Van Houten EEW , Doyley MM , Kennedy FE , Weaver JB , Paulsen KD . Initial in vivo experience with steady‐state subzone‐based MR elastography of the human breast. J Magn Reson Imaging. 2003;17:72‐85. 12500276 10.1002/jmri.10232

[51] McGarry M , Johnson CL , Sutton BP , et al. Including spatial information in nonlinear inversion MR elastography using soft prior regularization. IEEE Trans Med Imaging. 2013;32:1901‐1909. 23797239 10.1109/TMI.2013.2268978 PMC4107367

[52] Doyley MM , Van Houten EEW , Weaver JB , et al. Shear modulus estimation using parallelized partial volumetric reconstruction. IEEE Trans Med Imaging. 2004;23:1404‐1416. 15554128 10.1109/TMI.2004.834624

[53] Doyley MM , Feng Q , Weaver JB , Paulsen KD . Performance analysis of steady‐state harmonic elastography. Phys Med Biol. 2007;52:2657‐2674. 17473343 10.1088/0031-9155/52/10/002

[54] Van Houten EE , Doyley MM , Kennedy FE , Paulsen KD , Weaver JB . A three‐parameter mechanical property reconstruction method for MR‐based elastic property imaging. IEEE Trans Med Imaging. 2005;24:311‐324. 15754982 10.1109/tmi.2004.842451

[55] Van Houten EE , Viviers DV , McGarry M , et al. Subzone based magnetic resonance elastography using a Rayleigh damped material model. Med Phys. 2011;38:1993‐2004. 21626932 10.1118/1.3557469 PMC3077935

[56] Perriñez PR , Kennedy FE , Van Houten EE , Weaver JB , Paulsen KD . Modeling of soft poroelastic tissue in time‐harmonic MR elastography. IEEE Trans Biomed Eng. 2009;56:598‐608. 19272864 10.1109/TBME.2008.2009928 PMC2857336

[57] Perriñez PR , Kennedy FE , Van Houten EE , Weaver JB , Paulsen KD . Magnetic resonance poroelastography: An algorithm for estimating the mechanical properties of fluid‐saturated soft tissues. IEEE Trans Med Imaging. 2010;29:746‐755. 20199912 10.1109/TMI.2009.2035309 PMC2865251

[58] McGarry M , Van Houten E , Johnson C , et al. Multiresolution MR elastography using nonlinear inversion. Med Phys. 2012;39:6388‐6396. 23039674 10.1118/1.4754649 PMC3477197

[59] McGarry M , Van Houten E , Perrinez P , Pattison A , Weaver J , Paulsen K . An octahedral shear strain‐based measure of SNR for 3D MR elastography. Phys Med Biol. 2011;56:N153‐N164. 21654044 10.1088/0031-9155/56/13/N02 PMC3172714

[60] Perreard I , Pattison A , Doyley M , et al. Effects of frequency‐and direction‐dependent elastic materials on linearly elastic MRE image reconstructions. Phys Med Biol. 2010;55:6801‐6815. 21030746 10.1088/0031-9155/55/22/013 PMC3776577

[61] Kallel F , Bertrand M . Tissue elasticity reconstruction using linear perturbation method. IEEE Trans Med Imaging. 1996;15:299‐313. 18215911 10.1109/42.500139

[62] Jiang J , Varghese T , Brace CL , et al. Young's modulus reconstruction for radio‐frequency ablation electrode‐induced displacement fields: A feasibility study. IEEE Trans Med Imaging. 2009;28:1325‐1334. 19258195 10.1109/TMI.2009.2015355 PMC2843513

[63] Doyley MM , Meaney PM , Bamber JC . Evaluation of an iterative reconstruction method for quantitative elastography. Phys Med Biol. 2000;45:1521‐1540. 10870708 10.1088/0031-9155/45/6/309

[64] Miga MI . A new approach to elastography using mutual information and finite elements. Phys Med Biol. 2003;48:467‐480. 12630742 10.1088/0031-9155/48/4/304

[65] Eskandari H , Salcudean SE , Rohling R , Ohayon J . Viscoelastic characterization of soft tissue from dynamic finite element models. Phys Med Biol. 2008;53:6569‐6590. 18978443 10.1088/0031-9155/53/22/018

[66] Eskandari H , Goksel O , Salcudean SE , Rohling R . Bandpass sampling of high‐frequency tissue motion. IEEE Trans Ultrason Ferroelectr Freq Control. 2011;58:1332‐1343. 21768018 10.1109/TUFFC.2011.1953

[67] Goksel O , Eskandari H , Salcudean SE . Mesh adaptation for improving elasticity reconstruction using the FEM inverse problem. IEEE Trans Med Imaging. 2013;32:408‐418. 23192522 10.1109/TMI.2012.2228664

[68] Honarvar M , Rohling R , Salcudean SE . A comparison of direct and iterative finite element inversion techniques in dynamic elastography. Phys Med Biol. 2016;61:3026‐3048. 27002372 10.1088/0031-9155/61/8/3026

[69] Baghani A , Salcudean S , Honarvar M , Sahebjavaher RS , Rohling R , Sinkus R . Travelling wave expansion: A model fitting approach to the inverse problem of elasticity reconstruction. IEEE Trans Med Imaging. 2011;30:1555‐1565. 21813354 10.1109/TMI.2011.2131674

[70] Samani A , Bishop J , Plewes DB . A constrained modulus reconstruction technique for breast cancer assessment. IEEE Trans Med Imaging. 2001;20:877‐885. 11585205 10.1109/42.952726

[71] Karimi H , Fenster A , Samani A . A novel fast full inversion based breast ultrasound elastography technique. Phys Med Biol. 2013;58:2219‐2233. 23475227 10.1088/0031-9155/58/7/2219

[72] Zhang Y , Hall LO , Goldgof DB , Sarkar S . A constrained genetic approach for computing material property of elastic objects. IEEE Trans Evol Comput. 2006;10:341‐357.

[73] Fu D , Levinson SF , Gracewski SM , Parker KJ . Non‐invasive quantitative reconstruction of tissue elasticity using an iterative forward approach. Phys Med Biol. 2000;45:1495‐1509. 10870706 10.1088/0031-9155/45/6/307

[74] Zhu Y , Hall TJ , Jiang J . A finite‐element approach for Young's modulus reconstruction. IEEE Trans Med. Imaging. 2003;22:890‐901. 12906243 10.1109/TMI.2003.815065

[75] Guo Z , You S , Wan X , Biani N . A FEM‐based direct method for material reconstruction inverse problem in soft tissue elastography. Comput Struct. 2010;88:1459‐1468.

[76] Eskandari H , Salcudean SE , Rohling R , Bell I . Real‐time solution of the finite element inverse problem of viscoelasticity. Inverse Probl. 2011;27:1‐16. 085002.

[77] Albocher U , Oberai AA , Barbone PE , Harari I . Adjoint‐weighted equation for inverse problems of incompressible plane‐stress elasticity. Comput Methods Appl Mech Eng. 2009;198:2412‐2420.

[78] Barbone PE , Rivas CE , Harari I , Albocher U , Oberai AA , Zhang Y . Adjoint‐weighted variational formulation for the direct solution of inverse problems of general linear elasticity with full interior data. Int J Numer Methods Eng. 2010;81:1713‐1736.

[79] Zhang Y , Oberai AA , Barbone PE , Harari I . Solution of the time harmonic viscoelastic inverse problem with interior data in two dimensions. Int J Numer Methods Eng. 2012;92:1100‐1116.

[80] Albocher U , Barbone P , Richards M , Oberai A , Harari I . Approaches to accommodate noisy data in the direct solution of inverse problems in incompressible plane strain elasticity. Inverse Probl Sci Eng. 2014;22:1307‐1328. 25383085 10.1080/17415977.2013.872100 PMC4222193

[81] Honarvar M , Sahebjavaher RS , Salcudean SE , Rohling R . Sparsity regularization in dynamic elastography. Phys Med Biol. 2012;57:5909‐5927. 22955065 10.1088/0031-9155/57/19/5909

[82] Honarvar M , Sahebjavaher R , Sinkus R , Rohling R , Salcudean SE . Curl‐based finite element reconstruction of the shear modulus without assuming local homogeneity: Time harmonic case. IEEE Trans Med Imaging. 2013;32:2189‐2199. 23925367 10.1109/TMI.2013.2276060

[83] Manduca A , Muthupillai R , Rossman P , Greenleaf JF , Ehman RL . Local wavelength estimation for magnetic resonance elastography In: Proceedings of the International Conference on Image Processing, 1996. Vol. 3. Lausanne, Switzerland: IEEE; 1996:527‐530.

[84] Knutsson H , Westin CF , Granlund G . Local multiscale frequency and bandwidth estimation In: Proceedings of the IEEE International Conference Image Processing, ICIP‐94, Vol. 1. Austin, TX, USA: IEEE; 1994:36‐40.

[85] Braun J , Buntkowsky G , Bernarding J , Tolxdorff T , Sack I . Simulation and analysis of magnetic resonance elastography wave images using coupled harmonic oscillators and Gaussian local frequency estimation. Magn Reson Imaging. 2001;19:703‐713. 11672629 10.1016/s0730-725x(01)00387-3

[86] McGee KP , Lake D , Mariappan Y , et al. Calculation of shear stiffness in noise dominated magnetic resonance elastography data based on principal frequency estimation. Phys Med Biol. 2011;56:4291‐4309. 21701049 10.1088/0031-9155/56/14/006 PMC3144863

[87] Johnson CL , Chen DD , Olivero WC , Sutton BP , Georgiadis JG . Effect of off‐frequency sampling in magnetic resonance elastography. Magn Reson Imaging. 2012;30:205‐212. 22055750 10.1016/j.mri.2011.09.017

[88] Romano AJ , Shirron JJ , Bucaro JA . On the noninvasive determination of material parameters from a knowledge of elastic displacements theory and numerical simulation. IEEE Trans Ultrason Ferroelectr Freq Control. 1998;45:751‐759. 18244226 10.1109/58.677725

[89] Romano AJ , Bucaro JA , Ehman RL , Shirron JJ . Evaluation of a material parameter extraction algorithm using MRI‐based displacement measurements. IEEE Trans Ultrason Ferroelectr Freq Control. 2000;47:1575‐1581. 18238703 10.1109/58.883546

[90] Oliphant TE , Mahowald JL , Ehman RL , Greenleaf JF . Complex‐valued quantitative stiffness estimation using dynamic displacement, measurements, local inversion of conservation of momentum In: Ultrasonics Symposium, 1999. Proceedings IEEE, Vol. 2: Caesars Tahoe, NV, USA: IEEE; 1999:1641‐1644.

[91] Oliphant TE , Manduca A , Ehman RL , Greenleaf JF . Complex‐valued stiffness reconstruction for magnetic resonance elastography by algebraic inversion of the differential equation. Magn Reson Med. 2001;45:299‐310. 11180438 10.1002/1522-2594(200102)45:2<299::aid-mrm1039>3.0.co;2-o

[92] Manduca A , Oliphant TE , Dresner M , et al. Magnetic resonance elastography: Non‐invasive mapping of tissue elasticity. Med Image Anal. 2001;5:237‐254. 11731304 10.1016/s1361-8415(00)00039-6

[93] Manduca A , Lake DS , Kruse SA , Ehman RL . Spatio‐temporal directional filtering for improved inversion of MR elastography images. Med Image Anal. 2003;7:465‐473. 14561551 10.1016/s1361-8415(03)00038-0

[94] Sinkus R , Lorenzen J , Schrader D , Lorenzen M , Dargatz M , Holz D . High‐resolution tensor MR elastography for breast tumour detection. Phys Med Biol. 2000;45:1649‐1664. 10870716 10.1088/0031-9155/45/6/317

[95] Sinkus R , Tanter M , Xydeas T , Catheline S , Bercoff J , Fink M . Viscoelastic shear properties of in vivo breast lesions measured by MR elastography. Magn Reson Imaging. 2005;23:159‐165. 15833607 10.1016/j.mri.2004.11.060

[96] Okamoto R , Clayton E , Bayly P . Viscoelastic properties of soft gels: Comparison of magnetic resonance elastography and dynamic shear testing in the shear wave regime. Phys Med Biol. 2011;56:6379‐6400. 21908903 10.1088/0031-9155/56/19/014 PMC3178746

[97] McLaughlin J , Renzi D , Yoon JR , Ehman RL , Manduca A . Variance controlled shear stiffness images for MRE data. IEEE Int Symp on Biomedical Imaging: Macro to Nano. 2006:960‐963.

[98] Papazoglou S , Hamhaber U , Braun J , Sack I . Algebraic Helmholtz inversion in planar magnetic resonance elastography. Phys Med Biol. 2008;53:3147‐3158. 18495979 10.1088/0031-9155/53/12/005

[99] Riek K , Klatt D , Nuzha H , et al. Wide‐range dynamic magnetic resonance elastography. J Biomech. 2011;44:1380‐1386. 21295305 10.1016/j.jbiomech.2010.12.031

[100] Papazoglou S , Hirsch S , Braun J , Sack I . Multifrequency inversion in magnetic resonance elastography. Phys Med Biol. 2012;57:2329‐2346. 22460134 10.1088/0031-9155/57/8/2329

[101] Hirsch S , Guo J , Reiter R , et al. MR elastography of the liver and the spleen using a piezoelectric driver, single‐shot wave‐field acquisition, and multifrequency dual parameter reconstruction. Magn Reson Med. 2014;71:267‐277. 23413115 10.1002/mrm.24674

[102] Tzschätzsch H , Guo J , Dittmann F , et al. Tomoelastography by multifrequency wave number recovery from time‐harmonic propagating shear waves. Med Image Anal. 2016;30:1‐10. 26845371 10.1016/j.media.2016.01.001

[103] Elgeti T , Rump J , Hamhaber U , et al. Cardiac magnetic resonance elastography: Initial results. Invest Radiol. 2008;43:762‐772. 18923255 10.1097/RLI.0b013e3181822085

[104] Elgeti T , Laule M , Kaufels N , et al. Cardiac MR elastography: Comparison with left ventricular pressure measurement. J Cardiovasc Magn Reson. 2009;11:1‐10. 44. 19900266 10.1186/1532-429X-11-44 PMC2777142

[105] Sack I , Rump J , Elgeti T , Samani A , Braun J . MR elastography of the human heart: Noninvasive assessment of myocardial elasticity changes by shear wave amplitude variations. Magn Reson Med. 2009;61:668‐677. 19097236 10.1002/mrm.21878

[106] Kolipaka A , Araoz PA , McGee KP , Manduca A , Ehman RL . Magnetic resonance elastography as a method for the assessment of effective myocardial stiffness throughout the cardiac cycle. Magn Reson Med. 2010;64:862‐870. 20578052 10.1002/mrm.22467 PMC3035166

[107] Dresner MA , Rose GH , Rossman PJ , Muthupillai R , Manduca A , Ehman RL . Magnetic resonance elastography of skeletal muscle. J Magn Reson Imaging. 2001;13:269‐276. 11169834 10.1002/1522-2586(200102)13:2<269::aid-jmri1039>3.0.co;2-1

[108] Basford JR , Jenkyn TR , An KN , Ehman RL , Heers G , Kaufman KR . Evaluation of healthy and diseased muscle with magnetic resonance elastography. Arch Phys Med Rehabil. 2002;83:1530‐1536. 12422320 10.1053/apmr.2002.35472

[109] Bensamoun SF , Ringleb SI , Littrell L , et al. Determination of thigh muscle stiffness using magnetic resonance elastography. J Magn Reson Imaging. 2006;23:242‐247. 16374878 10.1002/jmri.20487

[110] Ringleb SI , Bensamoun SF , Chen Q , Manduca A , An KN , Ehman RL . Applications of magnetic resonance elastography to healthy and pathologic skeletal muscle. J Magn Reson Imaging. 2007;25:301‐309. 17260391 10.1002/jmri.20817

[111] Chakouch MK , Charleux F , Bensamoun SF . Quantifying the elastic property of nine thigh muscles using magnetic resonance elastography. PloS One. 2015;10:1‐13. e0138873. 10.1371/journal.pone.0138873 PMC4580449 26397730

[112] Jamin Y , Boult JKR , Li J , et al. Exploring the biomechanical properties of brain malignancies and their pathologic determinants in vivo with magnetic resonance elastography. Cancer Res. 2015;75:1216‐1224. 25672978 10.1158/0008-5472.CAN-14-1997 PMC4384983

[113] Streitberger KJ , Reiss‐Zimmermann M , Freimann FB , et al. High‐resolution mechanical imaging of glioblastoma by multifrequency magnetic resonance elastography. PloS One. 2014;9:1‐9. e110588. 10.1371/journal.pone.0110588 PMC4206430 25338072

[114] Kruse S , Smith J , Lawrence A , et al. Tissue characterization using magnetic resonance elastography: Preliminary results. Phys Med Biol. 2000;45:1579‐1590. 10870712 10.1088/0031-9155/45/6/313

[115] McKnight AL , Kugel JL , Rossman PJ , Manduca A , Hartmann LC , Ehman RL . MR elastography of breast cancer: Preliminary results. AJR Am J Roentgenol. 2002;178:1411‐1417. 12034608 10.2214/ajr.178.6.1781411

[116] Siegmann KC , Xydeas T , Sinkus R , Kraemer B , Vogel U , Claussen CD . Diagnostic value of MR elastography in addition to contrast‐enhanced MR imaging of the breast–initial clinical results. Eur Radiol. 2010;20:318‐325. 19727753 10.1007/s00330-009-1566-4

[117] Garteiser P , Doblas S , Daire JL , et al. MR elastography of liver tumours: Value of viscoelastic properties for tumour characterisation. Eur Radiol. 2012;22:2169‐2177. 22572989 10.1007/s00330-012-2474-6

[118] Li S , Chen M , Wang W , et al. A feasibility study of MR elastography in the diagnosis of prostate cancer at 3.0 T. Acta Radiol. 2011;52:354‐358. 21498375 10.1258/ar.2010.100276

[119] Sahebjavaher RS , Nir G , Gagnon LO , et al. MR elastography and diffusion‐weighted imaging of ex vivo prostate cancer: Quantitative comparison to histopathology. NMR Biomed. 2015;28:89‐100. 25382459 10.1002/nbm.3203 PMC5478374

[120] Sahebjavaher RS , Nir G , Honarvar M , et al. MR elastography of prostate cancer: Quantitative comparison with histopathology and repeatability of methods. NMR Biomed. 2015;28:124‐139. 25395244 10.1002/nbm.3218

[121] Rouviére O , Dresner MA , Rossman PJ , Burgart LJ , Fidler JL , Ehman RL . MR elastography of the liver: Preliminary results. Radiol. 2006;240:440‐8. 10.1148/radiol.2402050606 16864671

[122] Chen J , Talwalkar JA , Yin M , Glaser KJ , Sanderson SO , Ehman RL . Early detection of nonalcoholic steatohepatitis in patients with nonalcoholic fatty liverdisease by using MR elastography. Radiol. 2011;259:749‐756. 10.1148/radiol.11101942 PMC3099044 21460032

[123] Serai SD , Towbin AJ , Podberesky DJ . Pediatric liver MR elastography. Dig Dis Sci. 2012;57:2713‐2719. 22569825 10.1007/s10620-012-2196-2

[124] Loomba R , Wolfson T , Ang B , et al. Magnetic resonance elastography predicts advanced fibrosis in patients with nonalcoholic fatty liver disease: A prospective study. Hepatol. 2014;60:1920‐1928. 10.1002/hep.27362 PMC4245360 25103310

[125] Xanthakos SA , Podberesky DJ , Serai SD , et al. Use of magnetic resonance elastography to assess hepatic fibrosis in children with chronic liver disease. J Pediatr. 2014;164:186‐188. 24064151 10.1016/j.jpeds.2013.07.050 PMC3872246

[126] Fehlner A , Behrens JR , Streitberger KJ , et al. Higher‐resolution MR elastography reveals early mechanical signatures of neuroinflammation in patients with clinically isolated syndrome. J Magn Reson Imaging. 2015;44:51‐58. 26714969 10.1002/jmri.25129

[127] Sandroff BM , Johnson CL , Motl RW . Exercise training effects on memory and hippocampal viscoelasticity in multiple sclerosis: A novel application of magnetic resonance elastography. Neuroradiol. 2017;59:61‐67. 10.1007/s00234-016-1767-x 27889837

[128] Wu T , Felmlee JP , Greenleaf JF , Riederer SJ , Ehman RL . Assessment of thermal tissue ablation with MR elastography. Magn Reson Med. 2001;45:80‐87. 11146489 10.1002/1522-2594(200101)45:1<80::aid-mrm1012>3.0.co;2-y

[129] Corbin N , Vappou J , Breton E , et al. Interventional MR elastography for MRI‐guided percutaneous procedures. Magn Reson Med. 2016;75:1110‐1118. 25846380 10.1002/mrm.25694

[130] Serai SD , Wallihan DB , Venkatesh SK , et al. Magnetic resonance elastography of the liver in patients status‐post fontan procedure: Feasibility and preliminary results. Congenit Heart Dis. 2014;9:7‐14. 24134059 10.1111/chd.12144 PMC4584140

[131] Wallihan DB , Podberesky DJ , Marino BS , Sticka JS , Serai S . Relationship of MR elastography determined liver stiffness with cardiac function after fontan palliation. J Magn Reson Imaging. 2014;40:1328‐1335. 24408379 10.1002/jmri.24496

[132] Pepin KM , Chen J , Glaser KJ , et al. MR elastography derived shear stiffness–a new imaging biomarker for the assessment of early tumor response to chemotherapy. Magn Reson Med. 2014;71:1834‐1840. 23801372 10.1002/mrm.24825 PMC4214143

[133] Jugé L , Doan BT , Seguin J , et al. Colon tumor growth and antivascular treatment in mice: Complementary assessment with MR elastography and diffusion‐weighted MR imaging. Radiol. 2012;264:436‐444. 10.1148/radiol.12111548 22692038

[134] Kruse SA , Rose GH , Glaser KJ , et al. Magnetic resonance elastography of the brain. Neuroimage. 2008;39:231‐237. 17913514 10.1016/j.neuroimage.2007.08.030 PMC2387120

[135] Sack I , Beierbach B , Hamhaber U , Klatt D , Braun J . Non‐invasive measurement of brain viscoelasticity using magnetic resonance elastography. NMR Biomed. 2007;21:265‐271. 10.1002/nbm.1189 17614101

[136] Klatt D , Hamhaber U , Asbach P , Braun J , Sack I . Noninvasive assessment of the rheological behavior of human organs using multifrequency MR elastography: A study of brain and liver viscoelasticity. Phys Med Biol. 2007;52:7281‐7294. 18065839 10.1088/0031-9155/52/24/006

[137] Sack I , Beierbach B , Wuerfel J , et al. The impact of aging and gender on brain viscoelasticity. Neuroimage. 2009;46:652‐657. 19281851 10.1016/j.neuroimage.2009.02.040

[138] Clayton E , Garbow J , Bayly P . Frequency‐dependent viscoelastic parameters of mouse brain tissue estimated by MR elastography. Phys Med Biol. 2011;56:2391‐2406. 21427486 10.1088/0031-9155/56/8/005 PMC3158029

[139] Riek K , Millward JM , Hamann I , et al. Magnetic resonance elastography reveals altered brain viscoelasticity in experimental autoimmune encephalomyelitis. Neuroimage Clin. 2012;1:81‐90. 24179740 10.1016/j.nicl.2012.09.003 PMC3757734

[140] Freimann FB , Müller S , Streitberger KJ , et al. MR elastography in a murine stroke model reveals correlation of macroscopic viscoelastic properties of the brain with neuronal density. NMR Biomed. 2013;26:1534‐1539. 23784982 10.1002/nbm.2987

[141] Munder T , Pfeffer A , Schreyer S , et al. MR elastography detection of early viscoelastic response of the murine hippocampus to amyloid *β* accumulation and neuronal cell loss due to Alzheimer's disease. J Magn Reson Imaging. 2018;47:105‐114. 28422391 10.1002/jmri.25741

[142] Green MA , Bilston LE , Sinkus R . In vivo brain viscoelastic properties measured by magnetic resonance elastography. NMR Biomed. 2008;21:755‐764. 18457350 10.1002/nbm.1254

[143] Murphy MC , Huston JIII , Jack CRJr , et al. Measuring the characteristic topography of brain stiffness with magnetic resonance elastography. PLoS One. 2013;8:1‐14. e81668. 10.1371/journal.pone.0081668 PMC3847077 24312570

[144] Arani A , Murphy MC , Glaser KJ , et al. Measuring the effects of aging and sex on regional brain stiffness with MR elastography in healthy older adults. Neuroimage. 2015;111:59‐64. 25698157 10.1016/j.neuroimage.2015.02.016 PMC4387012

[145] ElSheikh M , Arani A , Perry A , et al. MR elastography demonstrates unique regional brain stiffness patterns in dementias. Am J Roentgenol. 2017;209:403‐408. 28570101 10.2214/AJR.16.17455 PMC5597304

[146] Guo J , Hirsch S , Fehlner A , et al. Towards an elastographic atlas of brain anatomy. PloS One. 2013;8:1‐10. e71807. 10.1371/journal.pone.0071807 PMC3743755 23977148

[147] Hetzer S , Birr P , Fehlner A , et al. Perfusion alters stiffness of deep gray matter. J Cereb Blood Flow Metabol. 2018;38:116‐125. 10.1177/0271678X17691530 PMC5757437 28151092

[148] Johnson CL , McGarry MD , Gharibans AA , et al. Local mechanical properties of white matter structures in the human brain. Neuroimage. 2013;79:145‐152. 23644001 10.1016/j.neuroimage.2013.04.089 PMC3676712

[149] Johnson CL , Schwarb H , DJ McGarry M , et al. Viscoelasticity of subcortical gray matter structures. Hum Brain Mapp. 2016;37:4221‐4233. 27401228 10.1002/hbm.23314 PMC5118063

[150] Anderson AT , Van Houten EE , McGarry MD , et al. Observation of direction‐dependent mechanical properties in the human brain with multi‐excitation MR elastography. J Mech Behav Biomed Mater. 2016;59:538‐546. 27032311 10.1016/j.jmbbm.2016.03.005 PMC4860072

[151] Testu J , McGarry M , Dittmann F , et al. Viscoelastic power law parameters of in vivo human brain estimated by MR elastography. J Mech Behav Biomed Mater. 2017;74:333‐341. 28654854 10.1016/j.jmbbm.2017.06.027

[152] Hawley JR , Kalra P , Mo X , Raterman B , Yee LD , Kolipaka A . Quantification of breast stiffness using MR elastography at 3 tesla with a soft sternal driver: A reproducibility study. J Magn Reson Imaging. 2017;45:1379‐1384. 27779802 10.1002/jmri.25511 PMC5395339

[153] Wassenaar PA , Eleswarpu CN , Schroeder SA , et al. Measuring age‐dependent myocardial stiffness across the cardiac cycle using MR elastography: A reproducibility study. Magn Reson Med. 2016;75:1586‐1593. 26010456 10.1002/mrm.25760 PMC4666827

[154] Arani A , Glaser KL , Arunachalam SP , et al. In vivo, high‐frequency three‐dimensional cardiac MR elastography: Feasibility in normal volunteers. Magn Reson Med. 2017;77:351‐360. 26778442 10.1002/mrm.26101 PMC4947569

[155] Bensamoun SF , Robert L , Leclerc GE , Debernard L , Charleux F . Stiffness imaging of the kidney and adjacent abdominal tissues measured simultaneously using magnetic resonance elastography. Clin Imaging. 2011;35:284‐287. 21724121 10.1016/j.clinimag.2010.07.009

[156] Warner L , Yin M , Glaser KJ , et al. Noninvasive in vivo assessment of renal tissue elasticity during graded renal ischemia using MR elastography. Invest Radiol. 2011;46:509‐514. 21467945 10.1097/RLI.0b013e3182183a95 PMC3128234

[157] Low G , Owen NE , Joubert I , et al. Reliability of magnetic resonance elastography using multislice two‐dimensional spin‐echo echo‐planar imaging (SE‐EPI) and three‐dimensional inversion reconstruction for assessing renal stiffness. J Magn Reson Imaging. 2015;42:844‐850. 25537823 10.1002/jmri.24826 PMC4560097

[158] Lee CU , Glockner JF , Glaser KJ , et al. MR elastography in renal transplant patients and correlation with renal allograft biopsy: A feasibility study. Acad Radiol. 2012;19:834‐841. 22503893 10.1016/j.acra.2012.03.003 PMC3377786

[159] Streitberger KJ , Guo J , Tzschätzsch H , et al. High‐resolution mechanical imaging of the kidney. J Biomech. 2014;47:639‐644. 24355382 10.1016/j.jbiomech.2013.11.051

[160] Venkatesh SK , Wang G , Teo LL , Ang BW . Magnetic resonance elastography of liver in healthy Asians: Normal liver stiffness quantification and reproducibility assessment. J Magn Reson Imaging. 2014;39:1‐8. 24123300 10.1002/jmri.24084

[161] Goss B , McGee KP , Ehman E , Manduca A , Ehman RL . Magnetic resonance elastography of the lung: Technical feasibility. Magn Reson Med. 2006;56:1060‐1066. 17036283 10.1002/mrm.21053

[162] McGee KP , Hubmayr RD , Ehman RL . MR elastography of the lung with hyperpolarized 3HE. Magn Reson Med. 2008;59:14‐18. 18058936 10.1002/mrm.21465

[163] Mariappan YK , Glaser KJ , Hubmayr RD , Manduca A , Ehman RL , McGee KP . MR elastography of human lung parenchyma: Technical development, theoretical modeling and in vivo validation. J Magn Reson Imaging. 2011;33:1351‐1361. 21591003 10.1002/jmri.22550 PMC3098473

[164] Mariappan YK , Kolipaka A , Manduca A , et al. Magnetic resonance elastography of the lung parenchyma in an in situ porcine model with a noninvasive mechanical driver: Correlation of shear stiffness with trans‐respiratory system pressures. Magn Reson Med. 2012;67:210‐217. 21590723 10.1002/mrm.22976 PMC3158832

[165] Mariappan YK , Glaser KJ , Levin DL , et al. Estimation of the absolute shear stiffness of human lung parenchyma using 1h spin echo, echo planar MR elastography. J Magn Reson Imaging. 2014;40:1230‐1237. 24390975 10.1002/jmri.24479 PMC4019718

[166] Klatt D , Papazoglou S , Braun J , Sack I . Viscoelasticity‐based MR elastography of skeletal muscle. Phys Med Biol. 2010;55:6445‐6459. 20952814 10.1088/0031-9155/55/21/007

[167] Barnhill E , Kennedy P , Hammer S , Beek EJ , Brown C , Roberts N . Statistical mapping of the effect of knee extension on thigh muscle viscoelastic properties using magnetic resonance elastography. Physiol Meas. 2013;34:1675‐1698. 24254405 10.1088/0967-3334/34/12/1675

[168] Cheng S , Gandevia S , Green M , Sinkus R , Bilston L . Viscoelastic properties of the tongue and soft palate using MR elastography. J Biomech. 2011;44:450‐454. 21040923 10.1016/j.jbiomech.2010.09.027

[169] Green M , Sinkus R , Gandevia S , Herbert R , Bilston L . Measuring changes in muscle stiffness after eccentric exercise using elastography. NMR Biomed. 2012;25:852‐858. 22246866 10.1002/nbm.1801

[170] Qin EC , Sinkus R , Geng G , et al. Combining MR elastography and diffusion tensor imaging for the assessment of anisotropic mechanical properties. A phantom study. J Magn Reson Imaging. 2013;37:217‐226. 22987805 10.1002/jmri.23797

[171] Green M , Geng G , Qin E , Sinkus R , Gandevia S , Bilston L . Measuring anisotropic muscle stiffness properties using elastography. NMR Biomed. 2013;26:1387‐1394. 23640745 10.1002/nbm.2964

[172] Itoh Y , Takehara Y , Kawase T , et al. Feasibility of magnetic resonance elastography for the pancreas at 3T. J Magn Reson Imaging. 2016;43:384‐390. 26149267 10.1002/jmri.24995

[173] Shi Y , Glaser KJ , Venkatesh SK , Ben‐Abraham EI , Ehman RL . Feasibility of using 3D MR elastography to determine pancreatic stiffness in healthy volunteers. J Magn Reson Imaging. 2015;41:369‐375. 24497052 10.1002/jmri.24572 PMC4122650

[174] Sahebjavaher RS , Baghani A , Honarvar M , Sinkus R , Salcudean SE . Transperineal prostate MR elastography: Initial in vivo results. Magn Reson Med. 2013;69:411‐420. 22505273 10.1002/mrm.24268

[175] Kemper J , Sinkus R , Lorenzen J , Nolte‐Ernsting C , Stork A , Adam G . MR elastography of the prostate: Initial in‐vivo application. Röfo‐fortschritte Auf Dem Gebiet Der Röntgenstrahlen Und Der Bildgebenden Verfahren. 2004;176:1094‐1099. 10.1055/s-2004-813279 15346284

[176] Sahebjavaher RS , Frew S , Bylinskii A , et al. Prostate MR elastography with transperineal electromagnetic actuation and a fast fractionally encoded steady‐state gradient echo sequence. NMR Biomed. 2014;27:784‐794. 24764278 10.1002/nbm.3118

[177] Charité Universitätsmedizin Berlin . BIOQIC‐Apps. https://bioqic‐apps.charite.de. Accessed March 20, 2018.

[178] Sinkus R , Tanter M , Catheline S , et al. Imaging anisotropic and viscous properties of breast tissue by magnetic resonance‐elastography. Magn Reson Med. 2005;53:372‐387. 15678538 10.1002/mrm.20355

[179] Tweten DJ , Okamoto RJ , Schmidt JL , Garbow JR , Bayly PV . Estimation of material parameters from slow and fast shear waves in an incompressible, transversely isotropic material. J Biomech. 2015;48:4002‐4009. 26476762 10.1016/j.jbiomech.2015.09.009 PMC4663187

[180] Chatelin S , Charpentier I , Corbin N , Meylheuc L , Vappou J . An automatic differentiation‐based gradient method for inversion of the shear wave equation in magnetic resonance elastography: Specific application in fibrous soft tissues. Phys Med Biol. 2016;61:5000‐5019. 27300107 10.1088/0031-9155/61/13/5000

[181] Schmidt J , Tweten D , Benegal A , et al. Magnetic resonance elastography of slow and fast shear waves illuminates differences in shear and tensile moduli in anisotropic tissue. J Biomech. 2016;49:1042‐1049. 26920505 10.1016/j.jbiomech.2016.02.018 PMC4851613

[182] Feng Y , Okamoto RJ , Namani R , Genin GM , Bayly PV . Measurements of mechanical anisotropy in brain tissue and implications for transversely isotropic material models of white matter. J Mech Behav Biomed Mater. 2013;23:117‐132. 23680651 10.1016/j.jmbbm.2013.04.007 PMC3752297

[183] Tweten D , Okamoto R , Bayly P . Requirements for accurate estimation of anisotropic material parameters by magnetic resonance elastography: a computational study. Magn Reson Med. 2017;78:2360‐2372. 28097687 10.1002/mrm.26600 PMC5513802

[184] Braun J , Braun K , Sack I . Electromagnetic actuator for generating variably oriented shear waves in MR elastography. Magn Reson Med. 2003;50:220‐222. 12815700 10.1002/mrm.10479

[185] Konofagou EE , Harrigan TP , Ophir J , Krouskop TA . Poroelastography: Imaging the poroelastic properties of tissues. Ultrasound Med Biol. 2001;27:1387‐1397. 11731052 10.1016/s0301-5629(01)00433-1

[186] Skovoroda AR , Lubinski L , Emelianov SY , O'Donnell M . Reconstructive elasticity imaging for large deformations. IEEE Trans Ultrason Ferroelectr Freq Control. 1999;46:523‐535. 18238453 10.1109/58.764839

[187] Oberai AA , Gokhale NH , Goenezen S , et al. Linear and nonlinear elasticity imaging of soft tissue in vivo: Demonstration of feasibility. Phys Med Biol. 2009;54:1191‐1207. 19182325 10.1088/0031-9155/54/5/006 PMC3326410

[188] Kaster T , Sack I , Samani A . Measurement of the hyperelastic properties of ex vivo brain tissue slices. J Biomech. 2011;44:1158‐1163. 21329927 10.1016/j.jbiomech.2011.01.019

[189] Fu Y , Chui C , Teo C , Kobayashi E . Elasticity imaging of biological soft tissue using a combined finite element and non‐linear optimization method. Inverse Probl Sci Eng. 2015;23:179‐196.

